# Silicon Nitride Coatings on Titanium for Cardiovascular Applications: Interface Engineering, Hemocompatibility, and Translational Challenges

**DOI:** 10.3390/ma19173668

**Published:** 2026-08-28

**Authors:** Oktawian Bialas

**Affiliations:** Materials Research Laboratory, Faculty of Mechanical Engineering, Silesian University of Technology, Konarskiego 18A Street, 44-100 Gliwice, Poland; oktawian.bialas@polsl.pl

**Keywords:** silicon nitride coatings, titanium, cardiovascular devices, interface engineering, hemocompatibility, physical vapor deposition, corrosion resistance, protein adsorption

## Abstract

**Highlights:**

- SiN_x_ coatings offer a promising strategy for improving titanium-based cardiovascular devices.
- Interface engineering governs coating adhesion, residual stress, and long-term stability.
- Deposition parameters critically determine the structural and biological performance of SiN_x_.
- Surface chemistry influences protein adsorption, platelet responses, and hemocompatibility.

**Abstract:**

Titanium and its alloys are widely used in cardiovascular devices because of their favorable mechanical properties, corrosion resistance, and biocompatibility. Nevertheless, their surfaces do not fully prevent nonspecific protein adsorption, platelet activation, thrombosis, bacterial colonization, or long-term degradation under physiological conditions. Silicon-nitride-based (SiN_x_) coatings represent a promising strategy for addressing these limitations by combining chemical stability, mechanical durability, hemocompatibility, and antibacterial activity. This review critically examines silicon-nitride-based (SiN_x_) coatings on titanium for cardiovascular applications, focusing on deposition technologies, interfacial phenomena, surface characteristics, and biological performance. Particular attention is given to physical vapor deposition parameters, coating adhesion, residual stresses, interfacial reactions, corrosion resistance, and mechanical stability. Relationships between surface chemistry, wettability, protein adsorption, platelet response, hemolysis, and cellular behavior are discussed, alongside the effects of static and dynamic testing conditions. SiN_x_ is also compared with Au, TiN, TiO_2_, ZrN, and silicon carbide-based coatings. Despite encouraging in vitro results, clinical translation remains limited by insufficient standardization, scarce long-term and flow-dependent data, and an incomplete understanding of degradation mechanisms. Future studies should integrate interface engineering with microfluidic models, standardized hemocompatibility testing, artificial intelligence-assisted optimization of process–structure–property–biological response relationships, and regulatory considerations to support the safe clinical translation of SiN_x_-coated cardiovascular devices.

## 1. Introduction

Cardiovascular diseases remain the leading cause of death globally, accounting for approximately 17.9 million deaths annually and 32% of worldwide mortality [1]. Cardiovascular diseases (CVDs) also remain a major cause of mortality and disease burden across Europe [2]. Despite significant medical advances over recent decades, this situation has not improved satisfactorily, necessitating continuous intensive development of treatment methods for chronic cardiovascular diseases [3]. Not every patient with cardiovascular disease is eligible for transplantation or will survive long enough for such procedures [4]. Therefore, there is a critical need to create and improve materials and devices that enable temporary or permanent function in compromised cardiac or circulatory states. The development of artificial organs represents one of the major achievements of biomedical engineering [5,6]. An additional benefit of advancing material selection is reducing hypersensitivity reactions in individuals sensitized to metals used in cardiac or other implants, a challenge often clinically indistinguishable from device infection and thus likely underrecognized in routine care. Evidence from systematic reviews highlights that differentiating hypersensitivity from cardiac implantable electronic device infection is difficult and may delay correct diagnosis and management, supporting the rationale for non-allergenic coatings or alternative materials when risk is suspected [7]. Devices primarily designed to enhance cardiac pumping capacity include Ventricular Assist Devices (VADs) and Total Artificial Heart (TAH) systems [8]. Although advances in pump design have improved the performance of ventricular assist devices, further progress increasingly depends on engineering the blood-contacting interface to reduce protein-mediated platelet activation, thrombosis, and flow-induced erythrocyte damage. While many device types address specific organ defects, the main limitations of current devices are hemolysis and thrombus formation due to clotting system activation resulting from blood-material contact [9,10]. These complications increase over time and can lead to serious conditions such as ischemic heart disease or stroke, with thrombotic events reported in TAH recipients [11,12].

Materials and surface modifications currently used in cardiovascular devices include titanium, sintered titanium, textured materials, titanium nitride (TiN) coatings, woven polyester, polyurethane, heparin, silicone, graphite powder, and polycarbonate [8]. Recent studies have identified Ti_3_Au-containing surface layers as a promising approach offering high hardness, wear resistance, and favorable cellular compatibility; however, direct protein adsorption and hemocompatibility data for Ti_3_Au remain unavailable [13,14]. Selected silicon-nitride-based thin films have demonstrated favorable platelet-adhesion behavior under specific in vitro conditions; however, these findings do not constitute direct evidence for SiN_x_-coated titanium under cardiovascular conditions [15]. Although TiN, TiO_2_, ZrN, Au, and silicon carbide-based coatings have demonstrated favorable mechanical, electrochemical, or biological properties, their hemocompatibility remains strongly dependent on surface composition, topography, wettability, testing conditions, and local hemodynamics. Si_3_N_4_ may offer a complementary combination of chemical stability, mechanical performance, and biologically active surface chemistry; however, evidence concerning its long-term behavior under cardiovascular conditions remains fragmented [16,17,18,19]. Moreover, the available evidence has not yet been comprehensively integrated across deposition technology, interfacial mechanics, surface chemistry, hemocompatibility, and long-term degradation. Therefore, this review critically examines SiN_x_ coatings deposited on titanium substrates, with particular emphasis on the relationships between deposition conditions, interfacial properties, hemocompatibility, and long-term stability in cardiovascular applications. This article provides a narrative review of studies addressing SiN_x_ coatings on titanium, with particular emphasis on coating characteristics, surface properties, and blood–material interactions. Because direct evidence concerning cardiovascular applications remains limited, studies on bulk Si_3_N_4_, related silicon-nitride-based coatings, and titanium cardiovascular surfaces were also considered where they provided relevant mechanistic or contextual information. For clarity, the evidence discussed in this review is interpreted according to its level of direct relevance. Direct evidence refers to SiN_x_-based coatings deposited on titanium and evaluated using blood-contacting or cardiovascularly relevant endpoints. Coating-specific indirect evidence includes SiN_x_, SiN_x_:H, or Si–N–O thin films investigated on non-titanium substrates or in non-cardiovascular experimental models, whereas contextual evidence includes bulk Si_3_N_4_, orthopedic and dental systems, Ti–Si–N materials, sensor architectures, and titanium cardiovascular surfaces without SiN_x_. These evidence categories are not treated as equivalent; indirect studies are used only to support mechanistic interpretation, establish processing–property relationships, or identify research gaps. Si_3_N_4_ is used for stoichiometric silicon nitride, particularly bulk ceramics or films for which the Si_3_N_4_ composition or phase has been explicitly identified. SiN_x_ denotes deposited silicon nitride films of unspecified or non-stoichiometric composition; SiN_x_:H denotes hydrogen-containing films; Si–N–O denotes oxygen-containing silicon–nitrogen films; and Ti–Si–N refers specifically to ternary titanium–silicon–nitrogen coatings.

## 2. Titanium-Based Materials in Cardiovascular Devices

Titanium and titanium alloys are among the most widely used metallic biomaterials because of their favorable combination of corrosion resistance, mechanical strength, relatively low density (approximately 4.5 g/cm^3^), fatigue resistance, and biocompatibility [20]. Their corrosion resistance in physiological environments is primarily associated with the spontaneous formation of a stable passive TiO_2_ layer. Additional advantages include their non-ferromagnetic behavior and relatively low thermal and electrical conductivity. However, titanium alloys generally exhibit poor intrinsic tribological properties and limited wear resistance, which may necessitate additional surface modification. Although reducing Young’s modulus is particularly important for implants interacting mechanically with bone, cardiovascular applications place greater emphasis on corrosion resistance, fatigue performance, surface stability, and hemocompatibility [21]. Ti6Al4V remains the most widely recognized and commonly used titanium alloy in biomedical engineering. Nevertheless, concerns regarding the possible release and biological effects of aluminum and vanadium have stimulated the development of alternative compositions. Ti6Al7Nb (ASTM F1295) [22] eliminates vanadium but retains aluminum, whereas Ti13Nb13Zr (ASTM F1713) [23] and Ti12Mo6Zr2Fe (ASTM F1813) [24] are free from both aluminum and vanadium [25,26]. Recent developments in Ti13Nb13Zr demonstrate that alloy composition and microstructural refinement can provide a favorable combination of strength and reduced stiffness. A nanostructured α- and β-phase microstructure with substructures smaller than 200 nm resulted in an ultimate tensile strength exceeding 990 MPa, fulfilling the requirements of ASTM F1713, while maintaining a Young’s modulus of 73 GPa [23]. The processing route may further influence the microstructure and fatigue behavior of Ti13Nb13Zr, as demonstrated for selectively laser-melted material [27]. Biological investigations additionally indicated reduced bacterial biofilm formation and enhanced osteoblast proliferation compared with commercially pure titanium Grade 4 and Ti6Al4V ELI, particularly for etched surfaces [26]. In cardiovascular devices, titanium and its alloys are used in components requiring high corrosion resistance, fatigue strength, biocompatibility, and long-term dimensional stability. Representative applications include polished blood-contacting and structural components of ventricular assist devices and total artificial hearts, as well as hermetic housings of pacemakers and implantable cardioverter-defibrillators. Titanium-based materials and surfaces have also been used or investigated in vascular stents, heart-valve components, and other blood-contacting cardiovascular systems [5,6,28,29].

**Figure 1:**
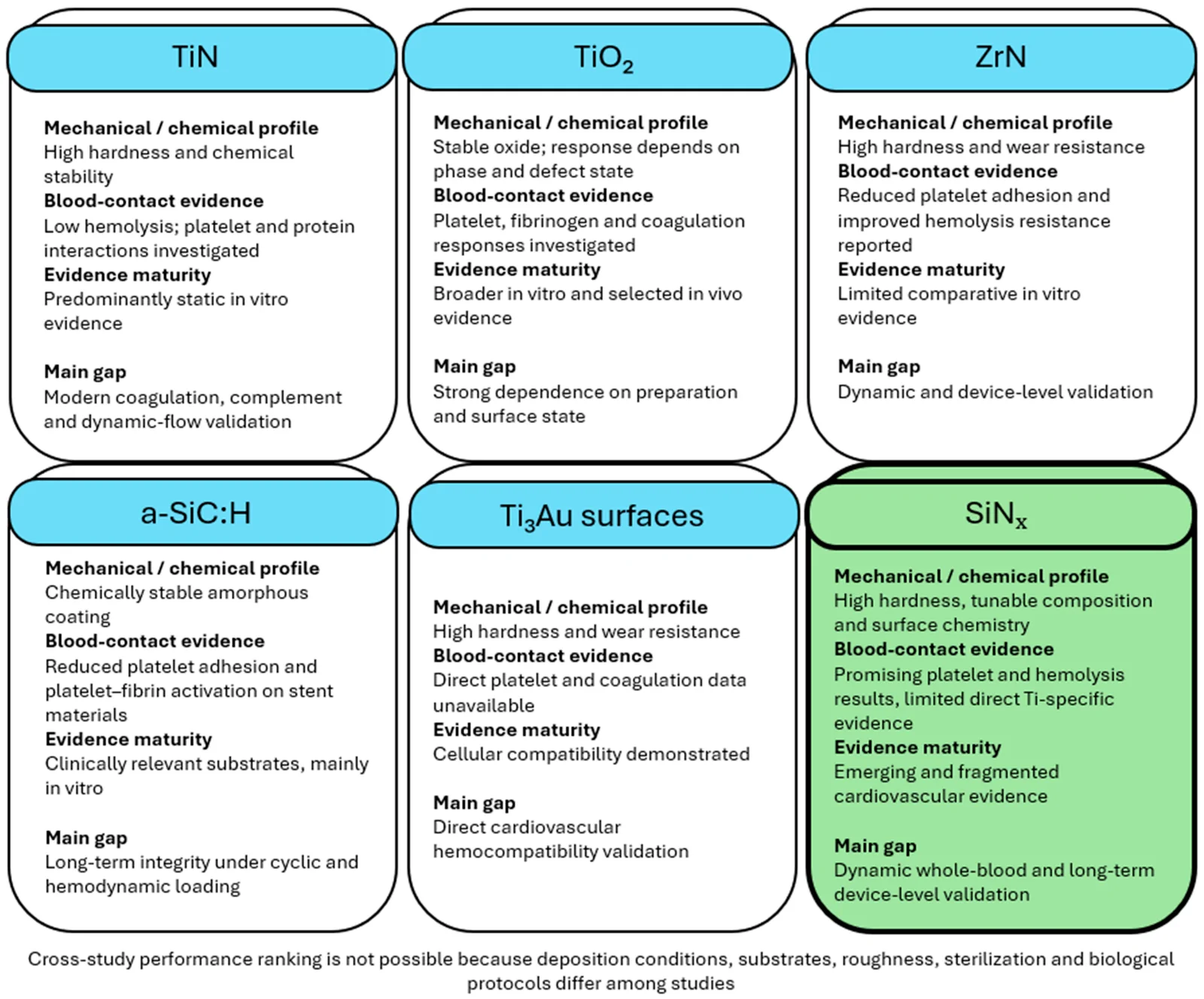
Comparative evidence landscape of representative surface-engineering strategies relevant to blood-contacting cardiovascular devices, TiN, TiO_2_, ZrN, a-SiC:H, Ti_3_Au-containing surfaces, and SiN_x_ are compared with respect to their principal mechanical and chemical characteristics, reported blood-contact response, maturity of the available evidence, and major remaining research gaps [13,14,15,33,34,35,36,37,38,39].

Despite their favorable bulk properties, unmodified titanium surfaces are not intrinsically thromboresistant. Immediately after contact with blood, the native TiO_2_ surface becomes covered by a dynamically evolving protein layer whose composition and conformation influence subsequent platelet adhesion and activation. In particular, fibrinogen–surface interactions depend not only on the chemical composition of TiO_2_, but also on its crystallographic structure, surface topography, wettability, and charge. Consequently, variations in the physicochemical state of the passive oxide layer may substantially affect the biological response to titanium-based blood-contacting components [30,31]. Engineered micro- and nanoscale titanium topographies can further modify hemocompatibility, illustrating that surface architecture and wettability are important determinants of the blood response [32]. These limitations have motivated the development of surface-engineering strategies aimed at controlling protein adsorption, platelet activation, endothelialization, and bacterial adhesion. The principal coating strategies investigated for this purpose, together with their reported advantages and limitations, are summarized in Figure 1.

Taken together, titanium-based materials provide favorable bulk mechanical properties, corrosion resistance, and long-term structural stability; however, their performance in blood-contacting applications is governed largely by the physicochemical state of the surface. As summarized in Figure 1, the available surface-engineering strategies can improve selected biological and functional properties, but their effectiveness remains strongly dependent on coating composition, processing conditions, surface defects, and the experimental model. These limitations justify further investigation of SiN_x_ as a potentially multifunctional coating for titanium-based cardiovascular components.

## 3. Biomedical Properties of Silicon Nitride: Bulk Ceramics and Thin-Film Coatings

### 3.1. Bulk Si_3_N_4_ as a Biomedical Reference Material

Silicon nitride (Si_3_N_4_) has emerged as a promising non-oxide bioceramic due to its favorable mechanical properties, chemical stability, biocompatibility, and biological activity. In contrast to many conventional implant ceramics, Si_3_N_4_ has been reported to inhibit bacterial proliferation while supporting the physiological activity of eukaryotic cells, including cell proliferation, differentiation, and osteogenic responses. This unusual combination of properties has supported its clinical use in intervertebral fusion devices and its broader investigation for orthopedic and dental implants, bone tissue engineering, antimicrobial and antiviral applications, and medical diagnostic components [16,17,40,41]. The biological behavior of Si_3_N_4_ is governed not only by its bulk composition and predominantly covalent bonding, but also by the physicochemical state of its surface. At the nanometer scale, Si_3_N_4_ surfaces may exhibit off-stoichiometric composition, lattice defects, partial oxidation, and the presence of Si–N, Si–O, and nitrogen-containing surface groups. During interactions with aqueous biological environments, this surface chemistry may provide biologically relevant silicon- and nitrogen-containing species, including silicic acid (H_4_SiO_4_), NH_4_^+^, NO_3_^−^, and N_2_. These species have been associated with cellular signaling, osteogenic differentiation, tissue integration, and antibacterial activity [16,42]. However, most available evidence concerning the biomedical activity of Si_3_N_4_ originates from bulk sintered ceramics, powders, composites, and bone-oriented experimental models. These effects cannot be transferred directly to thin SiN_x_ coatings deposited on titanium, because their biological response additionally depends on film stoichiometry, surface oxidation, phase constitution, roughness, wettability, defects, and deposition conditions. Thus, the established biomedical properties of bulk Si_3_N_4_ provide a biological rationale for its use as a coating, whereas its performance in cardiovascular blood-contacting applications requires separate evaluation under coating-specific and hemodynamically relevant conditions [15,35,43].

At the cellular level, Si_3_N_4_ should not be regarded as a biologically inert ceramic. Experimental studies have demonstrated favorable adhesion and proliferation of mammalian cells, together with enhanced mineralization and osteogenic differentiation. In a Si_3_N_4_-reinforced gelatin/chitosan cryogel system, pre-osteoblastic cells exhibited increased proliferation, mineral deposition, and upregulation of osteogenic genes under both static and cyclic loading conditions [40]. Bioactive Si_3_N_4_ surfaces have also been associated with apatite formation and cellular responses supporting bone integration [42,44,45]. Although these findings are derived mainly from bone-oriented experimental models, they demonstrate that the biological response to Si_3_N_4_ is actively shaped by its surface chemistry rather than by its bulk properties alone [16,17,46]. Surface modulation of Si_3_N_4_ has likewise been shown to alter its physicochemical and biological behavior, further emphasizing that the exposed surface state is a key determinant of biological performance [47]. Another distinctive feature of Si_3_N_4_ is its ability to suppress bacterial colonization while maintaining favorable responses of mammalian cells. Si_3_N_4_-containing systems have demonstrated reduced bacterial adhesion and biofilm formation against both Gram-positive and Gram-negative bacteria, including *Staphylococcus aureus* and *Escherichia coli*, without eliminating their cytocompatible and osteogenic properties [40,44,47]. Proposed mechanisms include the formation of ammonia and ammonium species during surface hydrolysis, changes in the local pH and osmotic balance, and the generation of reactive nitrogen species capable of disrupting bacterial membranes and metabolic processes [16,42,47]. However, the relative contribution of these mechanisms remains dependent on surface stoichiometry, oxidation state, microstructure, and experimental conditions. Consequently, antibacterial activity should be considered a surface-dependent property rather than an invariant characteristic of every Si_3_N_4_ material [48].

### 3.2. Thin-Film-Specific Surface Evolution and Biological Response

Thin SiN_x_ coatings should be considered separately from bulk Si_3_N_4_ ceramics because their biological behavior is governed by a finite material reservoir and by a surface state that may evolve continuously after deposition. Film stoichiometry, density, thickness, oxygen incorporation, defects, and surface oxidation influence both the dissolution rate and the chemical species presented at the coating–fluid interface. Pettersson et al. [18] investigated SiN_x_ coatings with N/Si atomic ratios of approximately 0.3, 0.8, and 1.1 and reported dissolution rates of approximately 0.2–1.4 nm/day, with a tendency towards lower dissolution rates at higher nitrogen contents. The lower-nitrogen coatings additionally exhibited increased Si–O bond formation at the surface and within the dissolution region. These observations demonstrate that the aqueous stability of a SiN_x_ coating is composition-dependent and that the surface exposed to biological fluids may differ chemically from the as-deposited film. The coating may therefore simultaneously act as a barrier limiting substrate-ion release and as a slowly evolving interface whose chemistry changes during physiological exposure [18]. Thin-film biological regulation likewise cannot be interpreted simply as a scaled-down form of the bioactivity reported for bulk Si_3_N_4_. Marin et al. [49] directly compared PVD silicon-nitride coatings with different Si ratios and demonstrated composition-dependent biological responses. Nitrogen-rich coatings exhibited stronger antibacterial activity against *E. coli* and supported cellular proliferation, whereas silicon-rich coatings produced a greater mineralized-tissue response in the investigated mesenchymal-cell model. These findings indicate that the biological response of a deposited SiN_x_ film can be deliberately modified through its surface composition rather than being an intrinsic and composition-independent property of silicon nitride. However, the investigated coatings were deposited on silica substrates and evaluated in bone-oriented experimental models; consequently, these results provide mechanistic evidence for thin-film-specific regulation but not direct evidence of cardiovascular hemocompatibility [49].

For blood-contacting coatings, the most immediate regulatory pathway is expected to involve the evolving physicochemical surface and the subsequently formed protein layer. Surface oxidation and hydroxylation, chemical bonding, wettability, surface charge, roughness, and nanoscale defects influence competitive protein adsorption and protein conformation, which in turn regulate platelet adhesion and activation. This mechanism is supported by studies of sputtered silicon-nitride-based films showing that differences in chemical bonding and surface-energy components are accompanied by substantial differences in platelet adhesion. Consequently, the cardiovascular response to SiN_x_ should be interpreted as a coupled deposition–surface evolution–protein–cell process rather than as a direct consequence of the biological properties established for bulk Si_3_N_4_ [15,36].

For blood-contacting applications, the initial adsorption of plasma proteins and the subsequent adhesion and activation of platelets are strongly influenced by surface energy, wettability, chemical bonding, and topography [15,30]. Shi et al. [15] investigated magnetron-sputtered silicon-nitride-based films produced under different N_2_ and CF_4_ flow conditions and demonstrated a close relationship between their chemical bonding state, the polar and dispersive components of surface energy, and platelet adhesion. Si–N–O films were identified as promising candidates for the development of antithrombogenic surfaces, although the reported evidence was limited to in vitro platelet adhesion under specific experimental conditions [15]. These results indicate that favorable hemocompatibility is not an automatic consequence of the presence of silicon and nitrogen, but rather the outcome of controlled film composition and physicochemical surface properties.

Taken together, bulk Si_3_N_4_ and thin SiN_x_ coatings share a common chemical basis but should not be treated as biologically equivalent material forms. Bulk ceramics provide important evidence of cytocompatibility, osteogenic bioactivity, and antibacterial behavior, whereas thin-film performance additionally depends on coating stoichiometry, thickness, density, surface oxidation, defects, dissolution kinetics, and the deposition-dependent physicochemical state of the exposed surface [18,49,50]. For cardiovascular applications, the most relevant thin-film mechanisms are therefore the evolution of surface chemistry during exposure and its consequences for protein conditioning, platelet response, and vascular-cell interactions. This distinction provides the basis for the following section, which considers how deposition conditions and interface engineering determine the structure and stability of SiN_x_ coatings on titanium [15,16,17,51].

## 4. Deposition, Characterization, and Stability of SiN_x_ Coatings on Titanium

**Figure 2:**
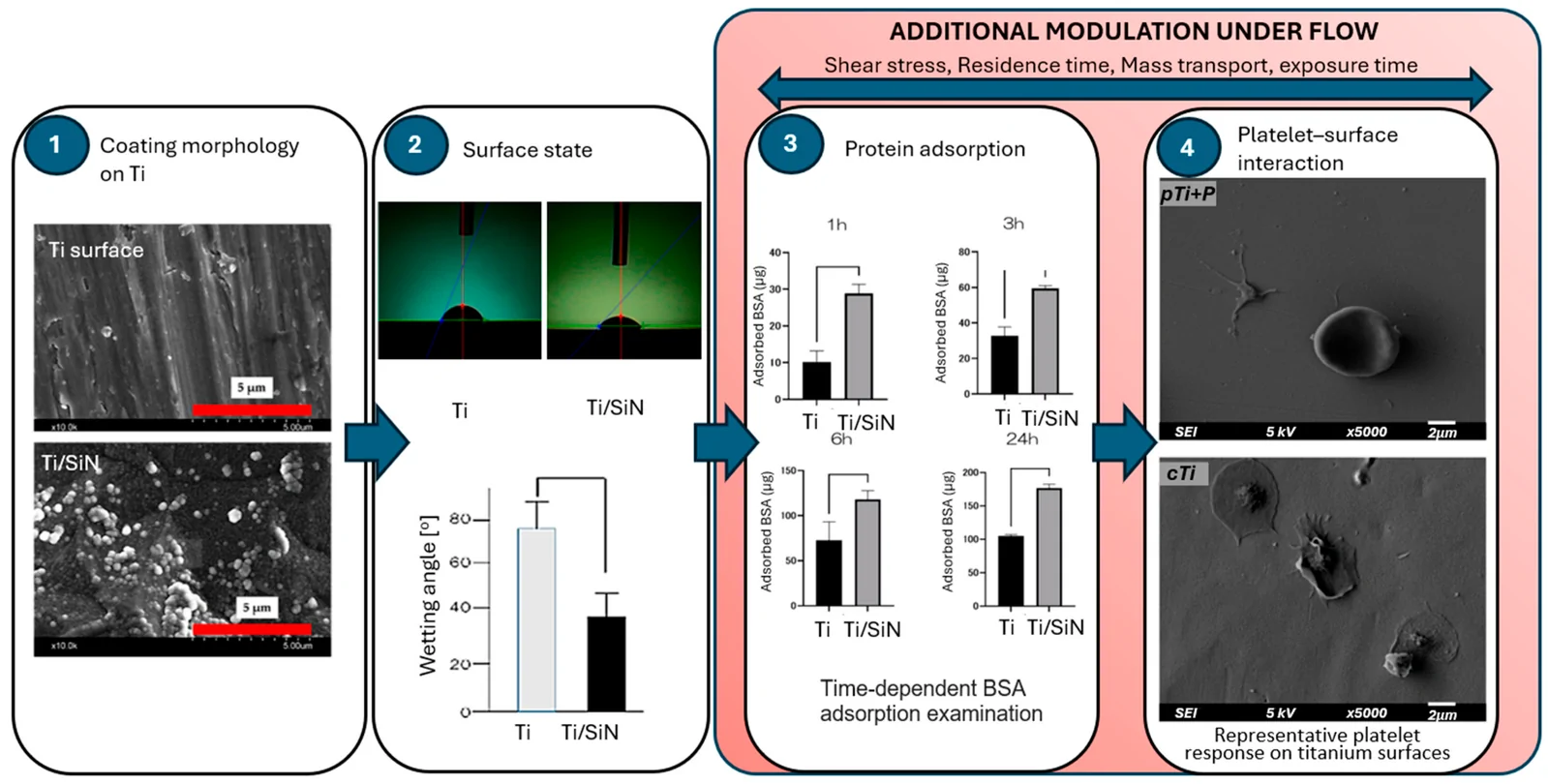
Evidence-based framework linking the surface state of SiN_x_-coated titanium to protein adsorption and blood-contacting responses. The scheme illustrates the progression from the titanium substrate and coating morphology (Panel 1), through the physicochemical state of the surface, represented here by water contact-angle measurements (Panel 2), to the formation of the biological interface, illustrated by time-dependent bovine serum albumin (BSA) adsorption (Panel 3), and the subsequent platelet response at the blood–material interface (Panel 4). Under device-relevant conditions, protein–surface and platelet–surface interactions may additionally be modulated by shear stress, residence time, mass transport, and exposure duration. Panels 1–3 were adapted from [35] and represent uncoated and SiN_x_-coated titanium. Panel 4 was adapted from [53] and provides a representative platelet response on titanium-based cardiovascular surfaces—it does not constitute direct evidence for SiN_x_-coated titanium. The original materials were cropped, rearranged, and relabeled by the author. Both source articles were distributed under the Creative Commons Attribution 4.0 International License (CC BY 4.0).

Silicon nitride coatings can be produced using both chemical vapor deposition (CVD) and physical vapor deposition (PVD) methods. Plasma-enhanced chemical vapor deposition enables relatively uniform coatings to be obtained at lower temperatures than conventional thermal CVD [52]. However, the use of gaseous precursors may lead to hydrogen incorporation and the formation of SiN_x_:H films. Tani et al. [35] deposited a ~200 nm silicon nitride layer, described by the authors as SiN, on commercially pure titanium using plasma chemical vapor deposition and confirmed the presence of silicon- and nitrogen-containing species on the modified surface. Their study demonstrated that a relatively thin SiN coating can modify the biological response of titanium without altering the bulk implant material [35]. Nevertheless, the experiments were conducted primarily in the context of dental implants and did not include long-term mechanical or cardiovascular testing. Figure 2 summarizes the links between SiN_x_ coating morphology, surface state, protein adsorption, and blood–material responses.

For metallic biomedical components, PVD methods, particularly magnetron sputtering, offer several practical advantages, including control overcoating thickness, relatively low processing temperatures, and the absence of reactive chemical precursors. Silicon nitride films may be deposited by sputtering a ceramic Si_3_N_4_ target in an Ar-containing atmosphere or by reactive sputtering of a silicon target in an Ar/N_2_ mixture. Because silicon nitride is electrically insulating, radio-frequency magnetron sputtering is commonly used to maintain stable plasma conditions. Pulsed magnetron sputtering and reactive high-power impulse magnetron sputtering have also been investigated to increase the ionization of the deposited species, improve coating density, and control residual stresses. Regardless of the selected method, the deposited layer should generally be described as SiN_x_ unless its stoichiometry and phase constitution have been directly confirmed. Sputtered coatings are frequently amorphous and may contain oxygen, hydrogen, fluorine, or an excess of either silicon or nitrogen [50,54,55,56]. The composition of the working atmosphere is one of the main factors governing SiN_x_ growth. Changes in the Ar/N_2_ ratio affect the target condition, nitrogen incorporation, deposition rate, chemical bonding, and the resulting film properties. Vila et al. [54] demonstrated that different sputtering atmospheres produced substantial differences in film stoichiometry and macroscopic properties. Signore et al. [55] similarly observed that the nitrogen fraction in the Ar/N_2_ atmosphere influenced the growth mechanism and chemical structure of SiN_x_ coatings deposited at room temperature [57]. This relationship is not necessarily linear. During reactive sputtering, an increase in nitrogen flow may promote target poisoning and reduce the sputtering rate, while the actual nitrogen content of the coating remains dependent on plasma conditions and the energy of the arriving species. Yau and Huang [58] reported changes in deposition rate, composition, hardness, and elastic modulus with increasing nitrogen flow, whereas Zhou et al. [59] showed that a higher sputtering current increased the deposition rate and surface roughness and promoted the transition from isolated islands towards a more continuous coating structure. Earlier biomedical studies also demonstrated the fabrication and evaluation of silicon-nitride coatings for total joint replacement applications, providing additional evidence that SiN_x_-based thin films can be engineered as functional implant surfaces [60].

Substrate temperature, bias voltage, target power, working pressure, and deposition time also influence the structure and stability of the coating. Increasing the substrate temperature generally enhances surface diffusion and may promote the formation of a denser film. However, a higher deposition temperature also increases the thermal contribution to the residual stress generated during cooling. For a simplified thin-film system, this contribution can be approximated as:

(1) $$|\sigma_{th}|\approx \frac{E_{f}}{1-\nu_{f}}|\alpha_{f}-\alpha_{s}||T_{d}-T_{0}|,$$

where $E_{f}$ and $\nu_{f}$ are the Young’s modulus and Poisson’s ratio of the film, $\alpha_{f}$ and $\alpha_{s}$ are the coefficients of thermal expansion of the film and substrate, $T_{d}$ is the deposition temperature, and $T_{0}$ is the reference or operating temperature. Equation (1) illustrates why the thermal expansion mismatch between SiN_x_ and titanium becomes increasingly important as the deposition temperature rises. It describes only the thermal component of stress, whereas the total residual stress also includes an intrinsic component generated during film nucleation, growth, ion bombardment, and defect incorporation [61].

**Figure 3:**
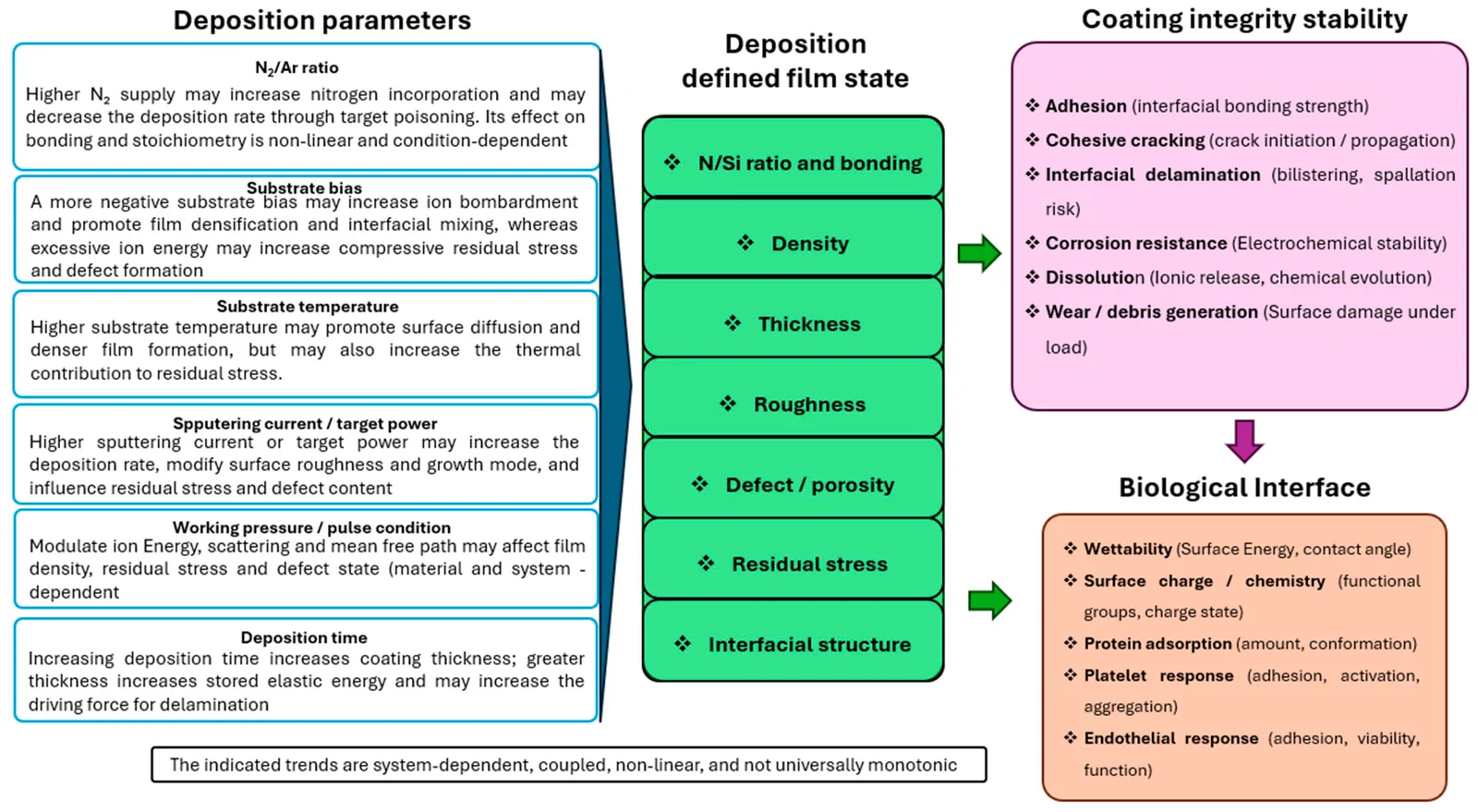
Schematic relationships between PVD parameters, the resulting SiN_x_ film state, coating integrity, and the biological interface. The N_2_/Ar ratio, substrate bias, substrate temperature, sputtering current or target power, working pressure and pulse conditions, and deposition time collectively influence film stoichiometry and bonding, density, thickness, roughness, defect/porosity density, residual stress, and interfacial structure. These characteristics govern coating adhesion, cracking and delamination behavior, corrosion resistance, dissolution, and wear, while simultaneously defining the physicochemical state presented to the biological environment. Changes in coating integrity during exposure may further modify wettability, surface chemistry, protein adsorption, platelet response, and endothelial interactions. The indicated trends are coupled, non-linear, and deposition-system dependent [50,54,55,58,59,61,63].

Ion bombardment induced by substrate bias can improve coating densification and interfacial mixing, but excessive ion energy may produce defects and high compressive stress. In reactive HiPIMS deposition, Schmidt et al. [50,62] demonstrated that the nitrogen-to-argon flow ratio, pulse conditions, target power, and substrate temperature could be used to control nitrogen incorporation and residual stress in amorphous SiN_x_ coatings. This is particularly important for thicker coatings because the elastic energy stored in the film increases with coating thickness and may eventually promote cohesive cracking or interfacial delamination [50]. The coupled relationships between the principal deposition parameters, the resulting film state, coating stability, and the biological interface are summarized in Figure 3.

A complete evaluation of SiN_x_ coatings requires complementary structural, chemical, and mechanical techniques. Surface and cross-sectional SEM can be used to evaluate morphology, thickness, continuity, and the presence of visible defects, while AFM or confocal microscopy provides quantitative information about surface topography and roughness. TEM, preferably combined with electron diffraction and local compositional analysis, is needed when the nanostructure of the coating–substrate interface is of interest. Conventional XRD may confirm the presence of crystalline phases, but the absence of diffraction peaks should not be interpreted as the absence of silicon nitride because thin sputtered SiN_x_ coatings are frequently X-ray amorphous. XPS, FTIR, and ToF-SIMS are therefore particularly useful for identifying Si–N, Si–O, Si–Si, and other nitrogen- or oxygen-containing surface groups. EDS can support the evaluation of elemental distribution but is not sufficient to determine the chemical bonding or exact stoichiometry of a thin SiN_x_ layer [15,54,59].

For illustrative purposes, the magnitude of the thermal contribution can be estimated for a representative SiN_x_–Ti system. Taking *E_f_* = 250 GPa, *ν_f_* = 0.25, *α_f_* = 2.5 × 10^−6^ K^−1^ for SiN_x_, *α_s_* = 8.6 × 10^−6^ K^−1^ for titanium, *T_d_* = 200 °C, and *T*_0_ = 37 °C, Equation (1) gives |*σ*_th_| ≈ 0.33 GPa. This value illustrates that thermal-expansion mismatch alone may generate stresses of several hundred MPa. However, it should not be interpreted as the total residual stress because intrinsic growth stress associated with ion bombardment, defect incorporation, and film densification may be of comparable or greater magnitude. Experimentally measured residual stresses in sputtered amorphous SiN_x_ coatings have been reported to vary approximately between 0.2 and 2.1 GPa depending on the deposition condition [50]. Equation (1) should therefore be interpreted as a first-order estimate of the thermal contribution to residual stress rather than as a complete stress model for a SiN_x_–Ti coating system. The approximation assumes a homogeneous, isotropic, linearly elastic film, a uniform temperature change, continuous interfacial bonding, and negligible stress relaxation during cooling. Real PVD SiN_x_ coatings may additionally exhibit intrinsic growth stress, compositional and density gradients, interfacial mixing, native or modified titanium oxide layers, local defects, and non-uniform thermal histories. Equation (1) also does not account for through-thickness stress gradients, multilayer or interlayer effects, substrate plasticity, or deposition-induced defects and local stress concentrations. Moreover, the thin-film approximation assumes that the substrate provides the dominant mechanical constraint. Consequently, the total residual stress and the associated delamination risk cannot be predicted from thermal-expansion mismatch alone and should be verified experimentally by residual-stress, adhesion, and interfacial-fracture measurements [61,62,63].

Mechanical characterization should include more than hardness measurement alone. Nanoindentation provides information about hardness and reduced elastic modulus [64,65]. The latter is related to the elastic properties of the tested material and the indenter according to:

(2) $$\frac{1}{E_{r}}=\frac{1-\nu_{f}^{2}}{E_{f}}+\frac{1-\nu_{i}^{2}}{E_{i}},$$

where $E_{r}$ is the reduced modulus, while $E_{f}$, $\nu_{f}$, $E_{i}$, and $\nu_{i}$ denote the Young’s moduli and Poisson’s ratios of the film and indenter, respectively. For a sufficiently thick and homogeneous material, this relationship allows the elastic modulus to be calculated from the indentation response. In thin SiN_x_ coatings, however, the measured value may be affected by the titanium substrate. The indentation depth should therefore remain sufficiently small relative to the coating thickness, and the results should be interpreted as those of a film–substrate system when the substrate contribution cannot be excluded [66].

The mechanical response of the coatings may be further compared using the ratios:

$$\frac{H}{E}\;\mathrm{and}\;\frac{H^{3}}{E^{2}},$$

where H and E represent coating hardness and elastic modulus. The H/E ratio is commonly associated with elastic strain tolerance, whereas $H^{3}/E^{2}$ is used as an approximate indicator of resistance to irreversible deformation [67]. These parameters are useful for comparing coatings produced under different conditions, although they cannot replace direct measurements of adhesion, fracture resistance, and wear. Scratch testing can reveal cohesive cracking, interfacial delamination, and complete coating failure, while tribological testing provides information about friction, wear, and the generation of coating debris [64]. Residual stresses can be determined from substrate curvature or diffraction-based methods. These properties should be considered together because a very hard coating may still exhibit limited durability if its fracture resistance is low or excessive residual stress reduces its adhesion. Shi et al. demonstrated that changes in the chemical bonding and surface properties of sputtered silicon-nitride-based films were accompanied by changes in their mechanical and tribological behavior [15].

Representative amorphous SiN_x_ coatings deposited by reactive HiPIMS exhibited hardness values of approximately 23–27 GPa and elastic moduli of approximately 240–265 GPa [50]. Taking representative values of *H* = 25 GPa and *E* = 250 GPa gives *H*/*E* ≈ 0.10 and *H*^3^/*E*^2^ ≈ 0.25 GPa. Using *E_f_* = 250 GPa, *ν_f_* = 0.25, and the conventional elastic constants of a diamond indenter, *E_i_* ≈ 1141 GPa and *ν_i_* ≈ 0.07, Equation (2) gives a reduced modulus *E_r_* of approximately 216 GPa. These values provide an order-of-magnitude reference rather than universal properties of SiN_x_, because the mechanical response varies with stoichiometry, density, residual stress, and deposition conditions [68].

The condition of the titanium surface before deposition is another important factor. Native TiO_2_, polishing residues, adsorbed carbon, and differences in substrate roughness may affect film nucleation and the development of the interface. Surface preparation affects not only coating nucleation and adhesion but also the physicochemical state of the final blood-contacting surface. Polishing and substrate roughness influence nucleation density, local coating thickness, defect formation, and mechanical interlocking, whereas the chemistry and thickness of the native TiO_2_ layer can modify interfacial bonding and local charge distribution. Plasma cleaning and ion etching may remove hydrocarbons and weakly bound oxide species, increase surface reactivity, and improve interfacial contact, but excessive treatment may also alter roughness or introduce near-surface defects [69]. Because these pretreatment-dependent changes can propagate through the deposited coating and influence wettability, protein adsorption, platelet interaction, and corrosion behavior, substrate preparation should be reported and controlled as part of the coating process rather than treated as a secondary experimental detail. Mechanical preparation followed by ultrasonic cleaning, plasma cleaning, or ion etching is therefore commonly performed before deposition. From a thermodynamic perspective, the ideal work of adhesion between the coating and the substrate can be expressed as:

(3) $$W_{ad}=\gamma_{{\mathrm{SiN}}_{x}}+\gamma_{Ti/TiN}-\gamma_{{\mathrm{SiN}}_{x}|Ti/TiN},$$

where $\gamma_{{\mathrm{SiN}}_{x}}$ and $\gamma_{Ti/TiN}$ are the surface energies of the coating and substrate or interlayer, respectively, and $\gamma_{{\mathrm{SiN}}_{x}|Ti/TiN}$ is their interfacial energy. A lower interfacial energy and a higher calculated work of adhesion indicate more favorable interfacial bonding. However, Equation (3) describes an ideal thermodynamic condition and does not account for contamination, pores, local defects, residual stress, or mechanical interlocking [70]. Experimental adhesion therefore cannot be inferred from surface energy alone.

Silicon incorporation into titanium oxide layers has also been considered as a method of modifying the substrate before the formation of the final functional coating. Rościszewska et al. [71] demonstrated that the incorporation of silicon into oxide layers formed on Ti13Nb13Zr altered their surface, mechanical, and biological properties. Although this system was produced by micro-arc oxidation rather than PVD, it supports the broader concept that the chemistry of the titanium surface can be deliberately modified to influence the properties of the subsequent interface.

A TiN interlayer may provide a more gradual transition between the metallic substrate and the ceramic SiN_x_ coating. Such a layer can improve chemical compatibility, redistribute stress, and protect the titanium surface during deposition [72,73,74]. At the same time, it introduces another interface at which cracking or delamination may occur. Ma et al. [75] investigated a TiN/Si_3_N_4_ multilayer system and showed that its mechanical response depended on film thickness, phase constitution, and mismatch strain. The system was deposited on stainless steel for sensor applications rather than on biomedical titanium, and its results cannot therefore be transferred directly to cardiovascular devices. Nevertheless, the study illustrates both the potential benefits and limitations of TiN/Si_3_N_4_ architectures and indicates why the interlayer thickness and residual stress require separate optimization [75]. Importantly, a TiN interlayer should not be regarded as intrinsically stress-relieving. A Ti/TiN/SiN_x_ architecture introduces two mechanically distinct interfaces, Ti/TiN and TiN/SiN_x_, and either may become the preferred crack or delamination path depending on the residual-stress state, layer thickness, crystallographic texture, elastic mismatch, and interfacial fracture resistance. Because crystalline TiN and predominantly amorphous SiN_x_ differ in elastic response, thermal expansion, and intrinsic growth stress, the multilayer architecture may generate local stress discontinuities even when the average coating stress remains acceptable. Reactive-magnetron-sputtered TiN/SiN_x_ multilayers investigated by Söderberg et al. [73] exhibited compressive residual stresses of approximately 1.3 ± 0.7 GPa, while the structure of the SiN_x_ interlayer changed with layer thickness, including epitaxial stabilization of a cubic SiN_x_-rich phase at sub-nanometer thicknesses. These observations demonstrate that interlayer thickness and interface structure can modify coherency stress and the mechanical response of the complete multilayer stack. Experimental deformation studies further show that multilayering can redirect rather than eliminate fracture. Parlinska-Wojtan et al. [74] investigated TiN/SiN_x_ multilayers deposited by reactive unbalanced magnetron sputtering at 200 °C and observed horizontal fractures along the SiN_x_ layers, vertical cracking associated with TiN grain boundaries, shear displacement of groups of bilayers, and delamination from the substrate during severe nanoindentation. Although these coatings were not deposited on biomedical titanium and the loading mode differed from cardiovascular service, the results demonstrate that TiN/SiN_x_ interfaces can act as preferential failure paths when local stresses exceed the corresponding interfacial fracture resistance. Consequently, the introduction of TiN should be evaluated at the level of the complete Ti/TiN/SiN_x_ stack rather than from the adhesion of the outer SiN_x_ layer alone.

Low-temperature PVD studies indicate that the TiN/SiN_x_ interface may differ fundamentally from the reaction layers observed during high-temperature joining. Patscheider et al. [72] prepared approximately four-monolayer Si_3_N_4_ overlayers on well-defined TiN(001) surfaces by reactive magnetron sputtering in Ar/N_2_ at room temperature and examined the resulting interfaces in situ by angle-resolved XPS. Si_3_N_4_ deposition produced a nitrogen-rich interfacial region and interfacial polarization, and the effect increased with more negative substrate bias. Importantly, no significant Ti–Si silicide formation was detected over the investigated bias range. The authors attributed the strengthened interface primarily to increased Ti–N coordination and polarization rather than to the formation of a thick silicide reaction layer. This provides direct evidence that low-temperature PVD interface chemistry cannot be inferred from high-temperature Ti–Si_3_N_4_ joining experiments. However, the model system consisted of ultrathin Si_3_N_4_ deposited on epitaxial TiN grown on MgO and should therefore be interpreted as mechanistic evidence rather than as direct validation of a biomedical Ti/TiN/SiN_x_ stack.

The possible formation of titanium nitrides and silicides at the SiN_x_–Ti interface also requires careful interpretation. TiN, Ti_5_Si_3_, TiSi, and other Ti–Si–N phases have been identified in high-temperature joining experiments involving titanium and bulk Si_3_N_4_. Lemus and Drew [76] observed TiN and titanium silicides after joining at temperatures above 1400 °C, while Tunckan et al. [77] identified a Ti_3_N_2_ reaction layer and a Ti_6_Si_3_N-containing region after heat treatment of a Si_3_N_4_–Ti joint at 1000 °C. These findings demonstrate that titanium can react with silicon nitride, but they do not prove that the same phases develop during PVD at temperatures of a few hundred degrees Celsius. In low-temperature coating systems, the interface may instead remain amorphous, partially oxidized, or limited to a very thin intermixed region. TiN, Ti_5_Si_3_, TiSi_2_, and related phases should therefore be regarded as possible interfacial products requiring experimental confirmation rather than as inherent components of every SiN_x_–Ti interface.

The resistance of the coating to delamination is determined by the balance between the energy available for crack propagation and the interfacial fracture resistance. For a simplified stressed thin film, the strain-energy release rate may be approximated as:

(4) $$G\approx Z\frac{\sigma^{2}h}{E_{f}^{\prime }},E_{f}^{\prime }=\frac{E_{f}}{1-\nu_{f}^{2}},G<G_{c},$$

where G is the energy release rate, Z is a dimensionless factor dependent on geometry and the delamination mechanism, σ is the residual stress, h is the coating thickness, and $E_{f}^{\prime }$ is the plane-strain modulus of the film. The coating remains stable when G is lower than the critical interfacial fracture energy $G_{c}$. This relationship shows why increasing the film thickness or residual stress may increase the driving force for delamination, even when the local chemical bonding at the interface is favorable [63]. The strong influence of residual stress on delamination can likewise be illustrated using Equation (4). For a 2 μm SiN_x_ coating with $E_{f}^{\prime }$ ≈ 267 GPa, increasing the residual stress from 0.2 to 2.1 GPa increases *G*/*Z* from approximately 0.30 to 33 J m^−2^. Thus, an approximately tenfold increase in residual stress produces an approximately two-orders-of-magnitude increase in the energetic driving force for delamination. The actual value of *G* additionally depends on the geometry-dependent factor Z, while coating stability requires comparison with the experimentally determined critical interfacial fracture energy *G_c_*. These numerical values should be regarded as an illustrative thin-film benchmark rather than a direct measurement of the SiN_x_–Ti interface. For a Ti/TiN/SiN_x_ architecture, this energy-balance concept applies separately to the Ti/TiN and TiN/SiN_x_ interfaces because each boundary has its own critical interfacial fracture energy. A TiN interlayer may therefore reduce the crack-driving force at one interface while transferring stress concentration or delamination susceptibility to the other.

In cardiovascular applications, coating stability should include resistance to mechanical loading, corrosion, prolonged immersion, and changes in surface chemistry. Coating density is also functionally important, as differences in the density of SiN_x_ coatings have been shown to influence the performance of coated implant systems [78]. A film that appears continuous in SEM may still contain nanoscale pores or defects through which the electrolyte can reach the titanium substrate. Potentiodynamic polarization and electrochemical impedance spectroscopy should therefore be performed after prolonged immersion and, where possible, after mechanical or hydrodynamic cycling. Changes in impedance, corrosion current, scratch-test response, and surface composition can then be used to distinguish initial coating quality from actual long-term stability. This is particularly important for SiN_x_ because partial surface oxidation and hydroxylation may alter wettability and protein interactions without causing visible coating failure [15,18,36].

Accordingly, deposition conditions, interfacial structure, mechanical properties, surface chemistry, and electrochemical stability should be evaluated as interconnected components of the same coating system. A favorable result obtained for one of these parameters does not alone demonstrate the suitability of the coating for blood-contacting devices. The following section therefore considers whether SiN_x_ coatings with controlled composition and stability provide measurable improvements in hemocompatibility and biological performance [15,78].

## 5. Hemocompatibility, Biological Performance, and Comparative Assessment

The biological response to a silicon nitride coating is determined not only by its nominal composition but also by the condition of the surface exposed to the physiological environment. Surface oxidation, hydroxylation, nanoscale roughness, charge, and the presence of residual contaminants can influence the first interactions with water, ions, and plasma proteins. This distinction is especially important for sputtered coatings, which are frequently amorphous and non-stoichiometric and should therefore be described as SiN_x_ rather than crystalline Si_3_N_4_ unless their composition has been confirmed. Consequently, biological results obtained for bulk silicon nitride cannot be directly assigned to a thin SiN_x_ coating without considering its processing history and surface chemistry.

Wettability is often used as an initial descriptor of the biological interface. For a liquid droplet on an ideal solid surface, the work of adhesion can be expressed using the Young–Dupré relationship [79]:

(5) $$W_{sl}=\gamma_{lv}\left(1+cos\;\theta \right),$$

where $W_{sl}$ is the work of adhesion between the solid and the liquid, $\gamma_{lv}$ is the liquid–vapor surface tension, and θ is the equilibrium contact angle. A decrease in θ therefore corresponds to an increase in the work of adhesion of water. Hydrophilic surfaces generally develop a more strongly associated interfacial water layer, whereas hydrophobic surfaces promote different orientations and conformational changes in adsorbed proteins. The relationship is not sufficiently direct, however, to conclude that a lower contact angle necessarily produces lower platelet adhesion. Contact-angle measurements are affected by roughness, surface heterogeneity, oxidation, and measurement conditions, while the response of blood is governed by the composition and biological activity of the adsorbed protein layer. A numerical example can be obtained directly from the SiN-coated titanium system reported by Tani et al. [35]. The water contact angle decreased from approximately 75° for uncoated titanium to approximately 35° for SiN-coated titanium. Taking the surface tension of water at room temperature as approximately 72.8 mN m^−1^, Equation (5) gives a work of adhesion of approximately 92 mJ m^−2^ for uncoated titanium and approximately 132 mJ m^−2^ for SiN-coated titanium. This calculation illustrates the substantial modification of the water–surface interaction produced by the coating. Nevertheless, the work of adhesion of water should be regarded as a physicochemical descriptor rather than a direct predictor of protein conformation, platelet adhesion, or platelet activation.

Protein adsorption begins within seconds of blood contact and precedes direct cellular interaction with the coating. Under simplified equilibrium conditions, the amount of adsorbed protein may be represented using a Langmuir isotherm adapted from the classical monolayer adsorption model [80,81]:

(6) $$\Gamma =\Gamma_{max}\frac{KC}{1+KC},$$

where Γ is the surface concentration of the adsorbed protein, $\Gamma_{max}$ is the maximum adsorption capacity, K is the adsorption equilibrium constant, and C is the protein concentration in the surrounding solution. Equation (6) is useful for illustrating the effects of concentration and surface affinity, but its assumptions are rarely satisfied in blood. Plasma contains competing proteins with different concentrations, diffusion coefficients, and affinities. Smaller and more abundant molecules may arrive first and subsequently be displaced by proteins with a greater affinity for the surface. Moreover, platelet response depends not only on the total amount of adsorbed fibrinogen but also on its orientation and the accessibility of platelet-binding domains [81]. Recent competitive adsorption experiments confirmed that platelet attachment may correlate more closely with the functional state of adsorbed fibrinogen than with its total surface concentration [82]. A quantitative application of Equation (6) to the currently available SiN-coated titanium data is not justified because determination of the maximum adsorption capacity and adsorption equilibrium constant requires concentration-dependent equilibrium adsorption measurements. The study by Tani et al. [35] reports time-dependent BSA adsorption rather than an equilibrium concentration series. Consequently, fitting a Langmuir model to these data would require assumptions that are not supported experimentally. Because of that, direct evidence concerning protein adsorption on SiN_x_-coated titanium remains limited. Studies of hydrogenated silicon nitride films produced by plasma-enhanced chemical vapor deposition showed that the N/Si ratio and resulting surface energy influenced film wettability and platelet interaction. Hydrophilic SiN_x_:H surfaces exhibited an acceptable in vitro blood response compared with low-temperature isotropic carbon, although the investigation was based primarily on static platelet experiments rather than complete cardiovascular-device testing [36]. Shi et al. [15] likewise demonstrated that changes in the chemical bonding, surface energy, and polar-to-dispersive surface-energy ratio of sputtered silicon-nitride-based films affected platelet adhesion. Their results suggest that the formation of Si–N–O-containing surface regions may contribute to the observed blood response. Nevertheless, the coatings also contained fluorine and oxygen, which makes it difficult to attribute the effect exclusively to Si–N bonding [15].

Fibrinogen is particularly relevant because its adsorption and conformational rearrangement can expose sequences recognized by platelet integrins, supporting platelet attachment, spreading, and aggregation. Albumin is generally considered less thrombogenic and may reduce access of adhesive proteins to the surface, although its protective effect depends on whether it remains stably adsorbed. Earlier work comparing titanium and gold already demonstrated that the amount and state of adsorbed fibrinogen depend strongly on surface composition and conditioning [83]. Therefore, comparisons between coatings should ideally include total protein adsorption, the relative adsorption of albumin and fibrinogen, and the biological availability of platelet-binding sites. Measuring only total protein concentration may conceal important differences in protein conformation.

Platelet adhesion provides useful information about the early thrombogenic response, but the number of attached platelets should not be interpreted in isolation. Platelet morphology, spreading area, pseudopodia formation, and expression or release of activation markers are equally important. A surface carrying a small number of strongly activated platelets may still initiate coagulation and thrombus development. A suitable testing strategy should therefore combine microscopic platelet counting with markers such as P-selectin, platelet factor 4, β-thromboglobulin, or activated glycoprotein IIb/IIIa. Thrombin generation, prothrombin fragment F1 + 2, thrombin–antithrombin complexes, and clotting-time measurements can then be used to evaluate whether platelet activation is accompanied by activation of the coagulation cascade. Complement activation should also be assessed, for example through markers such as C3a, C5a, or the terminal complement complex, because platelet and coagulation responses alone do not capture the complete blood–material interaction [84]. Accordingly, platelet adhesion should be regarded as one component of a broader hemocompatibility assessment that includes platelet activation, coagulation, complement activation, and whole-blood testing.

Evidence from related Ti–Si–N coatings is encouraging but must be interpreted separately from that of pure SiN_x_. Zhang et al. [85] deposited Ti–Si–N coatings on a titanium alloy using arc-enhanced magnetron sputtering. The films contained crystalline TiN and an amorphous Si_3_N_4_ phase. Selected deposition conditions improved corrosion resistance, prolonged clotting, reduced platelet-receptor activation, and supported vascular endothelial-cell spreading and endothelial nitric oxide synthase expression. These results support the use of silicon-containing nitride coatings in blood-contacting applications. They do not, however, isolate the contribution of silicon nitride from that of TiN, coating microstructure, residual stress, or surface roughness. Hemolysis represents another component of blood compatibility. In material screening, erythrocyte damage may result from extractable compounds, surface chemistry, sharp defects, or direct contact with an incompatible surface. In an operating cardiovascular device, hemolysis is also strongly influenced by fluid mechanics, including high shear stress, turbulent fluctuations, flow separation, and repeated passage through narrow gaps. A low hemolysis value obtained during static incubation therefore does not demonstrate that a coated device will be non-hemolytic under realistic flow conditions. Material-induced hemolysis and mechanically induced hemolysis should be treated as related but distinct problems [86].

**Figure 4:**
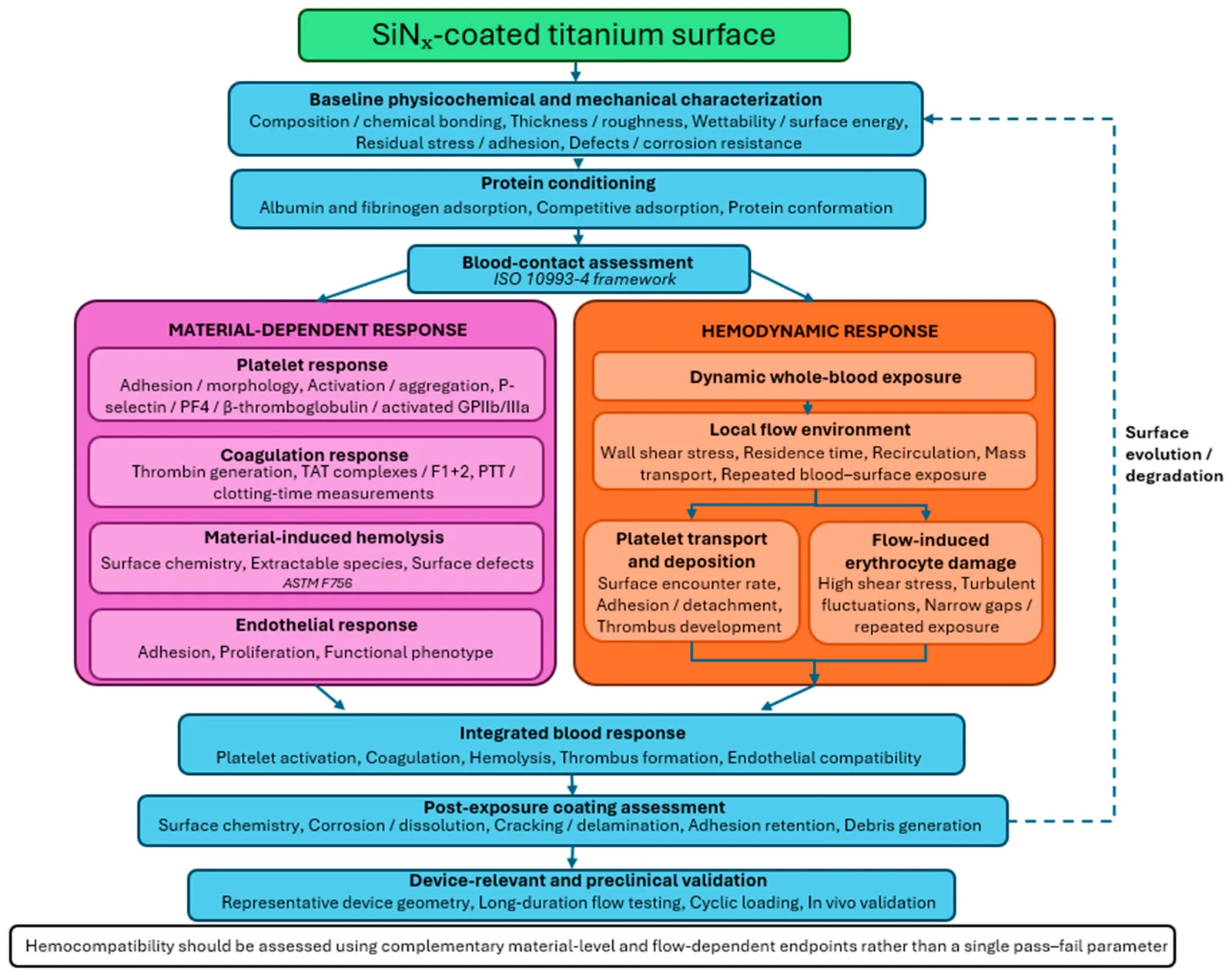
Proposed risk-based hemocompatibility evaluation workflow for SiN_x_-coated titanium surfaces intended for cardiovascular applications. The scheme integrates baseline surface characterization, protein conditioning, material-dependent blood responses, dynamic hemodynamic effects, post-exposure coating assessment, and device-relevant preclinical validation. Material-induced hemolysis is distinguished from flow-induced erythrocyte damage, and post-exposure characterization is included to assess surface evolution and coating integrity.

The biological evaluation should consequently follow a device-specific, risk-based approach. ISO 10993-4:2017, including Amendment 1:2025, provides the general framework for selecting tests for interactions between medical devices and blood, but does not prescribe a complete device-specific test program or universal acceptance criteria [87]. ASTM F756-17(2025) addresses material-induced hemolysis under defined laboratory conditions, but does not reproduce flow-induced erythrocyte damage occurring in operating cardiovascular devices [88]. ASTM F2382-24 provides a screening method for activation of the intrinsic coagulation pathway based on partial thromboplastin time in human plasma; it does not constitute a comprehensive assessment of coagulation, platelet activation, complement activation, or whole-blood thrombogenicity [89]. These standards should therefore be applied as complementary components of a device-specific hemocompatibility evaluation rather than as individual pass–fail tests. Test selection must reflect the type and duration of blood contact, expected flow conditions, and clinical function of the device [90]. Because no single assay is sufficient to establish cardiovascular hemocompatibility, material-level and flow-dependent endpoints should be integrated within a sequential, risk-based testing framework, as summarized in Figure 4.

Cytocompatibility is particularly relevant where the coated component is expected to contact the vascular wall or support endothelialization. Tani et al. [35] reported that an approximately 200 nm silicon nitride coating deposited on commercially pure titanium did not produce detectable cytotoxicity and promoted initial cell adhesion and osteogenic differentiation. The same surface reduced the attachment of *Staphylococcus aureus*. These results confirm that a thin silicon nitride coating can modify the biological response of titanium. However, the cells and experimental conditions were selected for dental and bone-contact applications. The results cannot be used as direct evidence of endothelial compatibility or cardiovascular safety.

More relevant vascular evidence is available for Ti–Si–N coatings. Zhang et al. [85,91] observed improved attachment, spreading, proliferation, and functional response of vascular endothelial cells on selected Ti–Si–N surfaces. Nevertheless, these observations require confirmation under flow, because endothelial-cell adhesion and phenotype are sensitive to wall shear stress [92]. Furthermore, a coating that supports endothelialization after implantation may still activate platelets during the period before a complete endothelial layer develops. Cytocompatibility and hemocompatibility must therefore be evaluated in parallel rather than treated as interchangeable properties.

Alloyed silicon nitride coatings provide another route for modifying biological performance. Skjöldebrand et al. [93] showed that chromium and niobium additions changed the dissolution behavior and cellular response of sputtered silicon nitride coatings. Certain compositions supported cell viability while reducing the dissolution rate. However, these films were deposited on CoCrMo with a CrN interlayer and were evaluated mainly in an orthopedic context. Adding metallic elements also changes the system from a nominally binary SiN_x_ coating into a multicomponent material whose corrosion products and long-term biological effects require separate investigation [94].

The antibacterial behavior of silicon nitride is an additional potential advantage over conventional passive coatings. Bulk Si_3_N_4_ and silicon-nitride-containing biomaterials have demonstrated activity against several clinically relevant microorganisms [42,44,95]. Evidence for thin coatings is more limited. Tani et al. [35] demonstrated reduced Staphylococcus aureus attachment on an approximately 200 nm silicon nitride coating deposited on commercially pure titanium, providing direct evidence that a thin silicon-nitride-based layer can impart antibacterial activity to a titanium surface. Marin et al. [49] also reported composition-dependent antibacterial activity of PVD SiN_x_ coatings against *E. coli*, although these coatings were deposited on silica rather than titanium. Importantly, neither study establishes antibacterial or anti-biofilm performance of SiN_x_-coated titanium under cardiovascularly relevant physiological conditions, including blood conditioning, prolonged immersion, or dynamic flow. Proposed mechanisms include surface reactions, local nitrogen-containing species, changes in pH, and oxidative or nitrosative stress. These mechanisms have already been discussed in Section 3 and do not need to be repeated in detail here. For thin coatings, the more important question is whether the antibacterial response is retained after surface oxidation, protein conditioning, sterilization, and prolonged immersion. Protein adsorption and blood conditioning may mask or modify the initially exposed SiN_x_ surface, changing surface charge, wettability, and the accessibility of chemically active sites involved in bacterial adhesion. Surface oxidation and prolonged immersion may further alter Si–N/Si–O surface chemistry and the release of nitrogen-containing species associated with the proposed antibacterial mechanisms. Sterilization may likewise modify surface chemistry, wettability, and defect state. However, these effects have not been systematically quantified for SiN_x_-coated titanium under cardiovascularly relevant conditions, and the persistence of antibacterial activity after sterilization, prolonged blood exposure, and protein conditioning remains unresolved. Accordingly, antibacterial activity demonstrated for bulk Si_3_N_4_, powders, or freshly prepared thin films cannot be assumed to persist after sterilization, protein conditioning, prolonged immersion, or blood exposure without direct coating-specific validation [96].

Most published hemocompatibility experiments on silicon-nitride-based surfaces have been performed under static or mildly agitated conditions. Such experiments are useful for initial material screening but do not reproduce the transport processes present in cardiovascular devices. Under flow, the local wall shear stress can be written as [97]:

(7) $$\tau_{w}=\mu {\dot{\gamma }}_{w},$$

where $\tau_{w}$ is the wall shear stress, μ is the dynamic viscosity, and ${\dot{\gamma }}_{w}$ is the wall shear rate. Wall shear stress affects platelet transport and adhesion, the stability of platelet aggregates, protein exchange, endothelial-cell behavior, and the mechanical loading of the coating [38,92,98]. Both very low shear regions associated with recirculation and high-shear regions near narrow gaps or disturbed jets may contribute to thrombosis through different mechanisms [98]. A simple numerical example illustrates the magnitude of this effect. Assuming an effective blood viscosity of 3.5 mPa·s, a wall shear rate of 100 s^−1^ corresponds to a wall shear stress of approximately 0.35 Pa, whereas increasing the shear rate to 1000 s^−1^ increases the wall shear stress to approximately 3.5 Pa. Thus, even without any change in coating chemistry, a tenfold change in the local shear rate produces a tenfold change in the mechanical stress acting on blood elements near the surface. These values are illustrative rather than device-specific because blood viscosity is shear-dependent and local shear rates depend on geometry and operating conditions.

The transport of a soluble species from the bulk fluid towards the surface may be approximated by [99]:

(8) $$J\approx k_{m}\left(C_{b}-C_{w}\right),$$

where J is the mass flux, $k_{m}$ is the mass-transfer coefficient, and $C_{b}$ and $C_{w}$ are the concentrations in the bulk fluid and near the wall, respectively. For an idealized laminar boundary layer, a Lévêque-type [99] scaling may be written as:

(9) $$k_{m}\propto {\left(\frac{D^{2}{\dot{\gamma }}_{w}}{L}\right)}^{1/3},$$

where D is the diffusion coefficient and L is a characteristic length. These relationships show that the biological response measured under flow cannot be attributed to surface chemistry alone. The Lévêque-type relationship also provides a useful scaling example for mass transport. If all other parameters remain unchanged, increasing the wall shear rate by one order of magnitude increases the mass-transfer coefficient by approximately 10^1/3^, i.e., about 2.15-fold. For the same bulk-to-wall concentration difference, Equation (8) therefore predicts an approximately 2.15-fold increase in the transport flux towards the surface. This example does not predict protein adsorption itself, but it demonstrates how a change in local hemodynamics can alter the rate at which soluble species are supplied to the coating. Flow changes the rate at which proteins, platelets, and reaction products reach or leave the surface. At the same time, Equations (8) and (9) do not determine whether a molecule will remain adsorbed, because adsorption kinetics, binding affinity, competitive displacement, and conformational changes still control the boundary condition at the coating surface. Numerical evaluation of Equations (7)–(9) is inherently device- and flow-condition-dependent rather than coating-specific. Wall shear stress, mass-transfer coefficients, diffusion distances, and near-wall concentration gradients depend on device geometry, blood rheology, flow rate, and local hemodynamic conditions. Assigning a single numerical value to a SiN_x_–Ti material system would therefore be physically misleading. These equations are instead intended to demonstrate how the biological response of the coating becomes coupled to mass transport and local hemodynamics under dynamic blood-contact conditions. The scarcity of dynamic studies remains one of the principal limitations of the current SiN_x_ evidence base. Parlak et al. [100] compared bulk alumina, zirconia, Si_3_N_4_, and SiC using cellular and blood-contact experiments that included dynamic bioreactor exposure. Although all tested ceramics exhibited acceptable cytocompatibility, monocrystalline SiC produced the most favorable platelet morphology in the direct comparison. This finding is relevant because it demonstrates that the ranking of candidate ceramic materials cannot be inferred from composition alone; however, the study concerned bulk materials rather than SiN_x_-coated titanium. Dynamic testing of other cardiovascular and biomaterial surfaces further illustrates the importance of flow conditions. Fallon et al. [38] demonstrated that platelet deposition under flow depended strongly on the dimensions of surface topographical features, indicating that conclusions obtained under static conditions may change when convective transport and shear are introduced. Van Oeveren et al. [101] compared several in vitro circulation models for thrombogenicity testing and showed that the experimental flow configuration itself influences the resulting blood response. Hönicke et al. [102] subsequently demonstrated a dynamic blood-compatibility approach for coronary stents using a pneumatic blood pump capable of generating physiologically relevant flow rates in small test chambers. Jamiolkowski et al. [103] likewise developed a whole-blood flow-loop system specifically for evaluating the thrombogenicity of medical devices and biomaterials. More recent studies further showed that incubation time, blood source, and the amount and geometry of the tested material can influence dynamic thrombogenicity results [104,105,106]. These studies do not provide direct validation of SiN_x_-coated titanium, but they demonstrate that surface chemistry, topography, wall shear stress, residence time, recirculation, blood source, and repeated blood–surface exposure form a coupled experimental system. Consequently, a favorable ranking obtained from static platelet-adhesion or hemolysis measurements should not be assumed to remain unchanged under physiologically relevant flow.

A comparative assessment of SiN_x_ should therefore consider the maturity of the available evidence as well as the biological results themselves. TiN is already used as a hard and chemically stable coating and has shown low hemolysis and acceptable platelet and protein interactions in early studies [33,107]. Its biological response nevertheless depends on oxidation, surface roughness, defects, and deposition conditions. TiO_2_ has a broader history of blood-contact investigation, including platelet, coagulation, and in vivo experiments. Different crystalline phases and defect structures can, however, produce different surface charges and protein interactions [30,37,108].

ZrN combines hardness and wear resistance with promising in vitro thromboresistance. Improved blood compatibility has been reported for ZrN deposited on NiTi, but the evidence cannot be transferred directly to SiN_x_-coated titanium devices [34]. Amorphous hydrogenated SiC is a particularly relevant comparator because it has been evaluated directly on stent materials. SiC-coated surfaces reduced platelet adhesion and platelet–fibrin activation relative to uncoated stainless steel and cobalt–chromium substrates [39]. Compared with these coatings, SiN_x_ offers the possible combination of mechanical stability, tunable dissolution, cytocompatibility, and antibacterial activity, but its cardiovascular evidence base is currently less mature.

Gold provides a useful reference because of its chemical nobility and long history in biomedical surface studies. Nevertheless, chemical nobility does not prevent fibrinogen adsorption [83]. Pure gold is also relatively soft, whereas intermetallic Ti_3_Au provides considerably higher hardness while retaining promising cellular compatibility [13,14]. Ti_3_Au should not be treated as biologically equivalent to a gold coating, however, because its phase composition, mechanical response, and surface chemistry are different. At present, to the best of the author’s knowledge, no sufficiently controlled head-to-head study has compared SiN_x_, TiN, TiO_2_, ZrN, SiC, and Au under identical deposition, roughness, sterilization, protein-conditioning, and flow conditions.

The current literature therefore supports SiN_x_ as a promising surface-engineering platform rather than as an already proven superior cardiovascular coating. Its principal strengths are the possibility of controlling composition and surface chemistry, compatibility with low-temperature deposition, favorable cellular response, and potential antibacterial activity. The main weakness is the limited number of studies performed directly on SiN_x_-coated titanium under physiologically relevant blood flow. Future studies should combine chemical and mechanical characterization with competitive protein adsorption, platelet and coagulation markers, endothelial-cell response, hemolysis, antibacterial testing, and dynamic whole-blood experiments [103,105,106]. Only such an integrated approach can determine whether the advantages observed for isolated biological endpoints translate into improved device-level performance.

The cited studies are representative rather than exhaustive; therefore, the number of publications supporting each individual conclusion is not reported as a formal evidence count. Such counts would require a systematic literature-search and study-selection methodology beyond the scope of this narrative review. Table 1 is intended as a concise cross-coating comparison whereas detailed quantitative deposition parameters for SiN_x_, including gas-flow conditions, substrate temperature, bias, pressure, residual stress, and representative film properties, are summarized separately in Table 2. Statements indicating that a parameter was not reported or evaluated refer specifically to the cited representative studies and do not imply the complete absence of such data from the broader literature.

The cited studies are representative rather than exhaustive. “Not reported” refers to the endpoints evaluated in the cited representative studies and does not necessarily indicate their complete absence from the broader literature. Direct comparison remains difficult because the available investigations differ in substrate composition, deposition method, coating thickness, surface roughness, sterilization, biological medium, and blood-contact conditions. The large number of interacting variables also makes conventional one-factor-at-a-time optimization inefficient. Deposition parameters, coating composition, surface state, mechanical integrity, protein adsorption, flow conditions, and biological outcomes form a coupled system. This provides the basis for the AI-assisted optimization and digital-twin approaches discussed in the following section.

## 6. AI-Assisted Optimization and Digital Twins for SiN_x_-Coated Cardiovascular Devices

**Figure 5:**
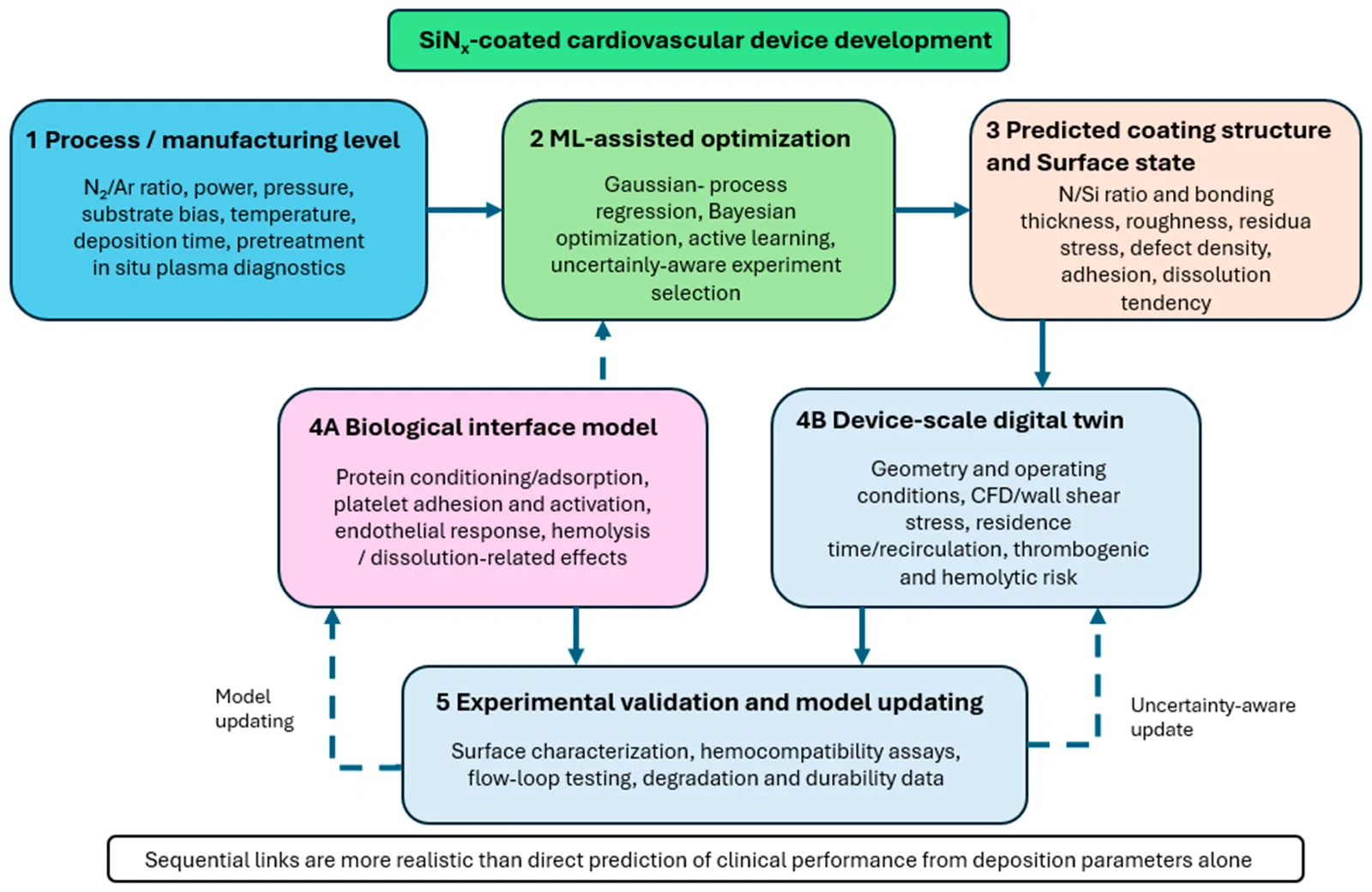
Hierarchical AI-assisted and digital-twin-enabled framework proposed for SiN_x_-coated cardiovascular devices. Deposition parameters and in situ process diagnostics are first related to coating composition and physicochemical properties using data-driven models. The experimentally characterized coating state is then linked to biological responses such as protein conditioning, platelet activation, endothelial interactions, hemolysis, and dissolution behavior. At the device level, these experimentally informed surface descriptors may be coupled with geometry- and flow-dependent hemodynamic models to estimate thrombogenic and hemolytic risk. Experimental characterization and flow-loop validation provide feedback for uncertainty-aware model updating and active-learning-guided experiment selection.

Unless otherwise stated, the AI-assisted and digital-twin applications discussed in this section should be regarded as prospective research directions rather than as validated approaches for SiN_x_-coated cardiovascular devices. The performance of SiN_x_-coated cardiovascular devices is governed by a complex chain of relationships linking deposition parameters, coating structure, surface properties, blood interactions, and device-scale hemodynamics. Conventional one-factor-at-a-time experiments are poorly suited to such a multidimensional system because they cannot efficiently identify interactions or trade-offs between mechanical, chemical, and biological properties. Artificial intelligence (AI), machine learning (ML), and digital-twin approaches may therefore support the rational development of these coatings. However, their present role should be interpreted cautiously. To the best of the authors’ knowledge, no validated framework currently integrates SiN_x_ deposition, coating characterization, dynamic hemocompatibility, and cardiovascular-device performance. Most available evidence originates from adjacent areas of thin-film processing, materials informatics, biomaterial screening, and cardiovascular modeling. A hierarchical view of the proposed AI-assisted and digital-twin-enabled development pathway for SiN_x_-coated cardiovascular devices is summarized in Figure 5.

The framework shown in Figure 5 is intentionally hierarchical rather than monolithic. In the near term, the most realistic strategy is not to predict clinical hemocompatibility directly from deposition parameters, but to build sequential links between processing, coating state, biological response, and device-scale hemodynamics. At the first level, data-driven models may predict coating composition, residual stress, thickness, roughness, and defect density from deposition parameters and in situ diagnostics. At the second level, the experimentally characterized surface state may be related to biological outcomes such as protein adsorption, platelet activation, endothelial response, and dissolution behavior. At the third level, these experimentally calibrated descriptors may be coupled with CFD-based or digital-twin-based device models to evaluate how the coating performs under realistic hemodynamic conditions.

### 6.1. AI-Assisted Optimization of SiN_x_ Coatings

ML methods can be used to identify relationships between processing conditions and the resulting material properties. For SiN_x_ coatings, relevant process variables may include target power, chamber pressure, Ar/N_2_ flow ratio, substrate bias and temperature, deposition time, pretreatment, and post-deposition processing. These parameters determine coating composition, thickness, chemical bonding, residual stress, hardness, adhesion, roughness, wettability, defect density, and degradation behavior [110]. In turn, the resulting surface state affects protein adsorption, platelet adhesion and activation, coagulation, hemolysis, endothelial-cell response, and bacterial attachment. AI-assisted optimization could connect these levels within a process–structure–property–biological response framework. A direct model predicting thrombogenicity from deposition settings would currently be difficult to justify because the available biological datasets are generally small and heterogeneous. A hierarchical strategy is more realistic. Separate models could first predict coating composition and physical properties from deposition conditions, followed by models relating the measured surface state to biological outcomes. This approach would preserve interpretable intermediate variables and help identify whether prediction uncertainty originates from manufacturing, coating characterization, or biological testing. The value of ML in materials development depends primarily on the consistency and physical relevance of the input descriptors and experimental data rather than on algorithmic complexity [111].

The feasibility of data-driven prediction during SiN_x_ deposition has already been demonstrated outside the biomedical field. Rachdi and Hofmann [112] correlated optical-emission spectra recorded during PECVD with SiN_x_ film thickness, refractive index, and Si–H and N–H bond densities. The developed models were subsequently used to identify alternative processing conditions producing similar films [112]. This study supports the use of plasma diagnostics for virtual metrology and process control. Nevertheless, the investigated coatings were intended for photovoltaic applications, and the predicted outputs did not include adhesion to titanium, long-term mechanical integrity, degradation, or biological performance [113]. Consequently, the model cannot be transferred directly to blood-contacting devices. Bayesian optimization and active learning appear particularly suitable for SiN_x_ coating development because they can guide the selection of subsequent experiments when data are limited and deposition and biological testing are expensive [114,115]. Instead of examining every possible combination of process parameters, a surrogate model identifies conditions expected either to improve coating performance or to reduce uncertainty in insufficiently explored regions. Similar closed-loop strategies combining deposition, characterization, and algorithmic selection have already been demonstrated for other thin-film materials [116,117]. Active-learning methods may therefore reduce the number of experiments required to define a useful SiN_x_ processing window [118,119].

For small experimental datasets typical of coating development, Gaussian-process regression is particularly suitable as a surrogate model for Bayesian optimization because it provides both a predicted property value and an associated uncertainty. For SiN_x_ deposition, the input vector could include N_2_/Ar ratio, target power, chamber pressure, substrate bias, substrate temperature, and deposition time, while the predicted outputs could include N/Si ratio, residual stress, hardness, defect density, dissolution rate, or coating adhesion. An acquisition function such as expected improvement or an upper-confidence-bound criterion can then balance exploitation of processing conditions predicted to provide favorable properties with exploration of insufficiently characterized regions. After the selected coating is deposited and characterized, the new observation is incorporated into the training set and the surrogate model is updated. Active learning follows a related iterative strategy, but the next experiment may instead be selected primarily according to prediction uncertainty, information gain, or proximity to a critical processing boundary. Such an approach could be particularly useful for identifying transitions between low- and high-residual-stress SiN_x_ deposition regimes or between dense and defect-prone coatings [114,115,118].

Specific thin-film process-prediction studies illustrate how these approaches could be transferred to SiN_x_ development. Rachdi and Hofmann used in situ optical-emission spectroscopy during PECVD SiN_x_ deposition to predict film thickness, refractive index, and Si–H/N–H bond densities, reporting maximum deviations of approximately 10.3%, 1.7%, and 35.8%, respectively. Shrivastava et al. [114] applied Bayesian optimization to sputter-deposited molybdenum films, using deposition power and chamber pressure to identify processing conditions satisfying residual-stress and sheet-resistance targets while reducing sensitivity to process fluctuations. Fébba et al. [117] combined optical-emission spectroscopy with Bayesian optimization in an autonomous sputtering workflow for Zn–Ti–N films and produced coatings with targeted cation compositions within approximately ±3.5% relative deviation. Although these examples were not developed for biomedical SiN_x_ coatings, they demonstrate experimentally validated routes for integrating plasma diagnostics, surrogate modeling, uncertainty-aware experiment selection, and closed-loop sputter-process control.

Optimization should nevertheless remain multi-objective [120]. Maximizing hardness or minimizing roughness does not necessarily improve hemocompatibility, while conditions supporting endothelialization may not simultaneously minimize early platelet activation. Relevant objectives could include reducing residual stress, coating defects, dissolution, platelet activation, thrombin generation, and hemolysis while maintaining adhesion, fatigue resistance, endothelial-cell functionality, and manufacturing reproducibility. Rather than identifying one universally optimal coating, the model should generate a set of Pareto-optimal solutions representing different compromises between these objectives [121]. Contact angle, surface energy, and total protein adsorption should be considered intermediate descriptors and not direct substitutes for dynamic blood compatibility.

### 6.2. Digital-Twin Framework for Coated Cardiovascular Devices

A digital twin is an evolving virtual representation of a physical system that is updated using experimental, manufacturing, or clinical observations [122]. A conventional CFD or finite-element simulation performed once and without subsequent updating should therefore be regarded as a computational model rather than a complete digital twin. In cardiovascular applications, digital twins have been proposed as frameworks combining mechanistic models, patient-specific information, and data-driven methods to support risk assessment and device or therapy selection [123].

For SiN_x_-coated cardiovascular devices, a digital twin could connect three related modeling levels. The first would represent the deposition process. Sensor data such as power, gas flow, chamber pressure, substrate temperature, deposition rate, and plasma-emission spectra could be used to estimate coating thickness, composition, bonding state, and residual stress. This manufacturing-level twin could detect process drift, assess whether a deposition batch remains within the validated processing window, and provide batch-specific properties for subsequent models. General frameworks for digital twins of plasma-processing systems have already been proposed, although their application to medical SiN_x_ coatings remains to be demonstrated [124].

The second level would describe the evolution of the coating after deposition. Sterilization, storage, immersion, protein conditioning, and cyclic device loading may change surface oxidation, dissolution, roughness, defect density, residual stress, and coating adhesion. These changes can modify the biological boundary condition even when macroscopic delamination is not observed. Experimentally determined degradation kinetics would therefore be required to update the predicted coating state over time.

The third level would represent device-scale hemodynamics. Patient- or device-specific geometry, blood rheology, inlet and outlet conditions, wall motion, and operating parameters could be used to calculate pressure, velocity, wall shear stress, residence time, recirculation, and exposure to damaging shear. Such models are increasingly applied to cardiovascular devices and patient-specific circulation [125]. However, conventional CFD generally treats the device wall as an inert no-slip boundary. If identical boundary conditions are assigned to uncoated titanium and SiN_x_-coated titanium, the simulation cannot distinguish between their protein adsorption, platelet attachment, dissolution, or coagulation behavior. A coating-sensitive digital twin would therefore require experimentally calibrated surface boundary conditions. These could include adsorption and desorption kinetics, surface-dependent platelet attachment and activation, dissolution rates, and the effects of shear stress and exposure time. Coupling these parameters with the transport relationships described in Section 5 would enable the model to represent interactions between local flow and the biological state of the coating. Without such coupling, CFD can identify unfavorable flow regions and estimate mechanically induced blood damage but cannot demonstrate that SiN_x_ is biologically superior to another surface. The most realistic near-term application is therefore a manufacturing and preclinical design twin rather than a continuously synchronized in vivo representation of the coating. Plasma diagnostics, surface characterization, electrochemical measurements, flow-loop experiments, and biological assays could update the model during coating and device development. Following implantation, the chemical state and protein coverage of a passive SiN_x_ coating cannot be measured directly without dedicated sensors and would have to be inferred indirectly from device signals, imaging, blood biomarkers, and degradation models [126].

Model credibility would require validation using independent deposition batches, blood donors, experimental campaigns, and flow conditions. Multiple measurements obtained from the same specimen should not be treated as independent training observations, as this would cause data leakage and overestimate predictive performance. Predictions should also include uncertainty estimates and indicate when a coating composition, biological protocol, or device geometry lies outside the model’s validated domain. Risk-informed frameworks such as ASME V&V 40 and the FDA guidance on computational modeling provide an appropriate basis for evaluating mechanistic components used in medical-device development [127]. Despite the conceptual attractiveness of this framework, the principal limitation is not algorithm availability but data scarcity and heterogeneity. Useful predictive datasets would need to link deposition parameters, process diagnostics, coating characterization, dynamic hemocompatibility, and device-scale observations for the same coating batches, but such integrated datasets are currently unavailable for SiN_x_-coated cardiovascular systems. Existing datasets are typically small, fragmented, and generated using different deposition platforms, substrates, sterilization procedures, and characterization protocols. Biological datasets introduce additional variability because platelet, coagulation, hemolysis, and endothelial endpoints may depend on donor-specific blood properties, exposure duration, assay format, and flow conditions. Long-term degradation, post-flow coating evolution, and negative or failed experimental outcomes are also rarely reported. These limitations increase the risks of overfitting, domain shift, and poor external validity. Future studies intended to support predictive modelling should therefore retain batch-level, specimen-level, donor-level, and experiment-level identifiers together with standardized metadata describing deposition, surface characterization, biological assays, and hemodynamic conditions.

AI and digital twins may ultimately connect manufacturing, coating characterization, biological testing, and hemodynamic modeling within a unified development framework. Their immediate value lies in prioritizing experiments, identifying sensitive parameters, improving batch reproducibility, and screening hypothetical device conditions. At present, however, the lack of standardized dynamic hemocompatibility data and long-term SiN_x_ degradation measurements represents a greater limitation than the availability of advanced algorithms. These approaches should consequently be presented as enabling research and design tools rather than as established evidence of clinical performance.

## 7. Conclusions

The available evidence does not yet demonstrate that SiN_x_ coatings provide superior cardiovascular performance compared with uncoated titanium or established surface-engineering systems. Their principal scientific interest arises from the possibility of tailoring composition, surface chemistry, mechanical properties, dissolution behavior, and biological interactions through deposition and interface engineering. However, these characteristics vary substantially with stoichiometry, deposition method, substrate condition, thickness, residual stress, oxidation, defects, and post-deposition exposure. Consequently, favorable results reported for an individual SiN_x_ composition or experimental model should not be generalized to silicon nitride coatings as a material class. Several limitations currently prevent definitive conclusions regarding cardiovascular performance. Direct studies of SiN_x_-coated titanium remain scarce, and much of the mechanistic evidence originates from bulk Si_3_N_4_, orthopedic coating systems, related Ti–Si–N materials, or non-titanium substrates. Biological investigations are dominated by static or mildly agitated in vitro assays, with limited standardized assessment of platelet activation, coagulation, complement, endothelial function, and whole-blood interactions under physiologically relevant flow. Long-term information concerning dissolution, corrosion, residual-stress evolution, fatigue, wear, cracking, interfacial delamination, sterilization effects, and coating integrity under combined mechanical and hemodynamic loading is also insufficient. Comparisons between coating systems are further complicated by differences in deposition conditions, substrate preparation, surface roughness, biological protocols, blood source, and experimental geometry. These limitations mean that current evidence supports SiN_x_ as a candidate surface-engineering strategy rather than as an established superior cardiovascular coating.

(i) Material preparation and interface engineering. Future studies should establish reproducible processing windows linking deposition parameters with SiN_x_ stoichiometry, chemical bonding, thickness, roughness, defect density, residual stress, adhesion, and dissolution behavior. Particular attention should be given to titanium surface preparation, native oxide condition, TiN or other interlayer architectures, interfacial fracture resistance, and the stability of the complete coating stack during sterilization, immersion, cyclic loading, and electrochemical exposure. Process optimization should therefore target a balanced combination of coating integrity, controlled surface chemistry, and manufacturing reproducibility rather than maximizing a single property such as hardness or wettability.
(ii) Biological and hemocompatibility testing. Biological evaluation should progress from isolated static endpoints towards standardized, complementary assessment of protein conditioning, platelet adhesion and activation, coagulation, complement activation, material-induced hemolysis, endothelial-cell response, and antibacterial behavior. These measurements should subsequently be integrated with dynamic whole-blood experiments performed under controlled and reported wall shear stress, residence time, recirculation, and mass-transport conditions. Material-induced blood damage should be distinguished from flow-induced erythrocyte and platelet damage, and coating integrity should be reassessed after dynamic exposure.
(iii) Preclinical and device-level verification. Coatings that satisfy the preceding material and biological criteria should then be evaluated in representative cardiovascular-device geometries under long-duration flow, cyclic mechanical loading, and clinically relevant operating conditions. Such studies should quantify both thrombogenic and hemolytic performance while monitoring coating degradation, delamination, debris formation, and changes in surface chemistry. Only after reproducible performance has been demonstrated in device-relevant flow loops and appropriate in vivo models will it be possible to determine whether SiN_x_ provides a clinically meaningful advantage over existing cardiovascular surface technologies. Across these three research branches, AI-assisted optimization and digital-twin approaches may support experimental prioritization, process control, and integration of coating and hemodynamic data. Their predictive value will, however, remain dependent on the availability of standardized, independent, and sufficiently large experimental datasets. At present, such computational tools should therefore be regarded as enabling methods for materials and device development rather than as substitutes for experimental or preclinical validation.

## Figures and Tables

**Table 1:** Multi-dimensional comparison of representative surface-engineering systems relevant to blood-contacting cardiovascular applications.

Surface System	Deposition/Formation Method, Substrate, and Thickness	Surface Roughness/Topography/Wettability	Platelet Adhesion/Activation/Coagulation Response	Hemolysis/Cellular Response	Mechanical/Electrochemical Stability, Dynamic/In Vivo Evidence, and Evidence Gap	**Representative Sources**
**SiN_x_ thin films**	PECVD SiN_x_:H films; plasma-CVD SiN coating on commercially pure Ti; representative Ti coating thickness ~200 nm	Increased surface topography after SiN deposition was observed by SEM on Ti; quantitative Ra was not reported in the representative Ti study	Composition-dependent platelet adhesion and activation; selected hydrophilic SiN_x_:H compositions showed reduced platelet interaction and acceptable hemocompatibility relative to LTI-C. No standardized activation grade is available	Low hemolytic activity reported for selected SiN_x_:H films; SiN-coated Ti showed no detectable cytotoxicity and supported initial cellular adhesion	**Integrity:** direct adhesion and residual-stress data were not reported for the representative SiN-coated Ti study; separate SiN_x_ thin-film studies reported residual stresses of ~0.2–2.1 GPa. Long-term cardiovascular corrosion protection remains insufficiently established. **Dynamic/in vivo:** no direct dynamic whole-blood or in vivo validation of SiN_x_-coated Ti. **Evidence gap:** long-term Ti/SiN_x_ integrity under cyclic hemodynamic loading remains unresolved	[18,35,36,53,109]
**TiN**	Chemical vapor deposition in the representative LVAD-oriented hemocompatibility study	Quantitative surface roughness and wettability were not assessed in the representative hemocompatibility study.	Protein adsorption and platelet retention were evaluated; modern platelet-activation markers were not reported, preventing assignment of a standardized activation degree	Very low hemolysis reported; cellular endpoints were not a principal part of the representative LVAD study	**Integrity:** adhesion, residual stress, and quantitative corrosion resistance were not principal endpoints of the representative hemocompatibility study. **Dynamic/in vivo:** evidence remains predominantly short-term and in vitro. **Evidence gap:** long-term cyclic coating integrity and in vivo cardiovascular validation remain insufficient	[33,107]
**TiO_2_**	Plasma immersion ion implantation and deposition (PIIID) and sputter deposition	Surface morphology and roughness characterized by AFM; roughness depended on film structure and processing, but no single representative Ra should be assigned across the investigated variants	Reduced platelet adhesion and favorable coagulation responses were reported for selected Ti–O films; no universal activation grade	Hemolysis was not the principal endpoint of the representative study	**Integrity:** adhesion and residual stress were not principal endpoints; electrochemical performance depended on oxide structure and processing. **Dynamic/in vivo:** in vivo implantation was reported for ~17–90 days. **Evidence gap:** long-term mechanical integrity under cyclic cardiovascular loading was not systematically quantified	[37]
**ZrN**	Magnetron sputtering on electropolished NiTi porous surface morphology with particles; ~2.5 μm coating thickness	Porous surface morphology with particles was observed, quantitative roughness and wettability were not reported in the representative study	Reduced platelet adhesion/improved thromboresistance relative to uncoated NiTi, standardized activation markers were not reported	Improved resistance to hemolysis relative to the uncoated substrate; cellular response not a principal endpoint	**Integrity:** adhesion and residual stress were not reported as principal endpoints. **Dynamic/in vivo:** no dynamic whole-blood or long-term in vivo coating-integrity validation. **Evidence gap:** cyclic-loading and device-level durability remain unresolved	[34]
**a-SiC:H**	Commercial/clinically relevant a-SiC:H-coated stainless-steel and Co–Cr stent surfaces, coating process not specified in the representative comparative study	Test probes were polished to provide comparable surface structures; quantitative roughness and wettability values were not provided in the representative comparative study	Markedly reduced platelet adhesion and platelet–fibrin activation; activation was rarely observed and most attached platelets remained in a resting morphology	Hemolysis and cellular-response endpoints were not evaluated in the representative platelet–thrombus comparison	**Integrity:** adhesion, residual stress, and quantitative corrosion resistance were not evaluated in the representative blood-contact comparison. **Dynamic/in vivo:** controlled low-shear flow testing was performed. **Evidence gap:** long-term coating integrity, cyclic deformation, and implantation stability were not investigated	[39]
**Au**	Gold reference surface; no deposited coating was evaluated in the representative adsorption study	Quantitative roughness and wettability were not characterized in the representative fibrinogen-adsorption study	Fibrinogen adsorption was evaluated; direct platelet activation and coagulation endpoints were not assessed	Hemolysis and cellular-response endpoints were not evaluated in the representative fibrinogen-adsorption study	**Integrity:** coating adhesion and residual stress not applicable to the reference surface; corrosion resistance was not an endpoint. **Dynamic/in vivo:** no dynamic or in vivo cardiovascular validation. **Evidence gap:** representative evidence is limited primarily to protein adsorption	[83]
**Ti_3_Au-containing surface layers**	Au electrodeposition followed by laser surface microalloying of Ti13Zr13Nb	Pre-coating Ti13Zr13Nb substrate: Ra = 0.46 ± 0.08 μm and Rz = 3.51 ± 0.83 μm; these values do not represent the final laser-microalloyed surface	Direct platelet-activation and coagulation data unavailable	No cytotoxic response observed in the investigated cellular model	**Integrity:** conventional coating adhesion and residual stress were not quantified for the laser-microalloyed layer; improved wear resistance was demonstrated. **Dynamic/in vivo:** direct cardiovascular flow and in vivo validation unavailable. **Evidence gap:** cardiovascular corrosion and hemocompatibility remain insufficiently characterized	[13,14]

**Table 2:** Process–structure–stability–biological response framework for SiN_x_ coatings relevant to titanium-based blood-contacting systems.

Deposition or Surface Parameter	Representative Numerical Context	Stability/Characterization Parameter	Relevant Equation	Biological Relevance/Evaluation
N_2_/Ar ratio and Si/N composition	N_2_/Ar flow ratios of approximately 0.28–0.30 produced N-rich amorphous SiN_x_ coatings; approximately 50 at.% N was reported [50]	XPS, FTIR, ToF-SIMS, EDS, phase assessment by XRD	-	Surface composition and chemical bonding affect wettability, protein adsorption, and platelet response
Substrate temperature and thermal mismatch	Illustrative SiN_x_–Ti calculation at 200 °C deposition temperature and 37 °C reference temperature gives a thermal-stress magnitude of approximately 0.33 GPa	Residual-stress measurements, coating morphology, adhesion	Equation (1)	Excessive residual stress may promote cracking or delamination and expose the underlying titanium
Pressure, bias voltage, and pulse energy	In rHiPIMS SiN_x_ coatings, increasing the negative substrate bias from −60 to −200 V increased the compressive residual stress from approximately 0.74 to 1.85 GPa at 400 mPa and from approximately 0.49 to 0.90 GPa at 600 mPa. Low residual stress (~0.2 GPa) was obtained using a pressure of 600 mPa, substrate temperatures below 200 °C, pulse energies below 2.5 Ws, and moderate negative bias voltages up to approximately −100 V [50]	Substrate-curvature measurements, XRD, SEM, adhesion testing	Equations (1) and (4)	Mechanical integrity during prolonged blood exposure and cyclic loading
Hardness and elastic modulus	Hardness approximately 23–27 GPa; elastic modulus approximately 240–265 GPa [50] representative *H*/*E* ≈ 0.10 and *H*^3^/*E*^2^ ≈ 0.25 GPa	Nanoindentation, tribological testing	Equation (2)	Wear resistance, debris formation, and preservation of the functional surface
Coating thickness and residual stress	For an illustrative 2 μm coating, increasing residual stress from 0.2 to 2.1 GPa increases *G*/*Z* from approximately 0.30 to 33 J m^−2^	Cross-sectional SEM/TEM, scratch testing, interfacial fracture assessment	Equation (4)	Long-term coating integrity and prevention of substrate exposure
SiN coating on titanium and wettability	Approximately 200 nm SiN coating on Ti [35] contact angle decreased from approximately 75° to approximately 35° calculated work of adhesion increased from approximately 92 to 132 mJ m^−2^	Contact-angle measurement, surface-energy analysis	Equation (5)	Initial water and protein interaction with subsequent platelet–surface response
Protein adsorption	Increased time-dependent BSA adsorption was reported for SiN-coated titanium [35] equilibrium Langmuir parameters were not determined	BSA/fibrinogen adsorption and protein-conformation assays	Equation (6)	Protein-layer composition and conformation influence platelet adhesion and activation
Blood-contact response under flow	No universal coating-specific numerical value; conditions depend on device geometry and operating regime	Flow loop, microfluidics, CFD, whole-blood testing	Equations (7)–(9)	Platelet adhesion and activation, coagulation, hemolysis, endothelial response, and thrombus formation

Numerical values obtained from SiN_x_ thin-film studies using non-titanium substrates are included as representative coating benchmarks and should not be interpreted as direct measurements of the SiN_x_–Ti interface. Values obtained directly for SiN-coated titanium are identified separately. The biological endpoints should be selected according to the intended cardiovascular device and blood-contact conditions.

## Data Availability

No new data were created or analyzed in this study. Data sharing is not applicable to this article.

## Abbreviations

The following abbreviations are used in this manuscript:

AFM	Atomic force microscopy
ASME	American Society of Mechanical Engineers
AI	Artificial intelligence
BSA	Bovine serum albumin
CVDs	Cardiovascular diseases
CVD	Chemical Vapor Deposition
CFD	Computational fluid dynamics
EDS	Energy-dispersive X-ray spectroscopy
FTIR	Fourier-transform infrared spectroscopy
FDA	U.S. Food and Drug Administration
HiPIMS	High-power impulse magnetron sputtering
ISO	International Organization for Standardization
ML	Machine learning
PECVD	Plasma-enhanced chemical vapor deposition
PVD	Physical vapor deposition
SEM	Scanning electron microscopy
TAH	Total artificial heart
TEM	Transmission electron microscopy
ToF-SIMS	Time-of-flight secondary ion mass spectrometry
VAD	Ventricular assist device
XPS	X-ray photoelectron spectroscopy
XRD	X-ray diffraction
